# Enhancing co-seismic landslide susceptibility, building exposure, and risk analysis through machine learning

**DOI:** 10.1038/s41598-024-54898-w

**Published:** 2024-03-11

**Authors:** Ajaya Pyakurel, Diwakar K.C., Bhim Kumar Dahal

**Affiliations:** 1 Department of Civil Engineering, IOE, Pulchowk Campus, TU, Lalitpur, Nepal; 2 Department of Civil and Environmental Engineering, University of Toledo, Toledo, OH 43606 USA; 3 Institute of Hazard, Risk and Resilience, Durham University, Durham, UK

**Keywords:** Co-seismic landslide, Machine learning, Susceptibility mapping, Exposure, Disaster risk management, Natural hazards, Engineering

## Abstract

Landslides are devastating natural disasters that generally occur on fragile slopes. Landslides are influenced by many factors, such as geology, topography, natural drainage, land cover, rainfall and earthquakes, although the underlying mechanism is too complex and very difficult to explain in detail. In this study, the susceptibility mapping of co-seismic landslides is carried out using a machine learning approach, considering six districts covering an area of 12,887 km^2^ in Nepal. Landslide inventory map is prepared by taking 23,164 post seismic landslide data points that occurred after the 7.8 MW 2015 Gorkha earthquake. Twelve causative factors, including distance from the rupture plane, peak ground acceleration and distance from the fault, are considered input parameters. The overall accuracy of the model is 87.2%, the area under the ROC curve is 0.94, the Kappa coefficient is 0.744 and the RMSE value is 0.358, which indicates that the performance of the model is excellent with the causative factors considered. The susceptibility thus developed shows that Sindhupalchowk district has the largest percentage of area under high and very high susceptibility classes, and the most susceptible local unit in Sindhupalchowk is the Barhabise municipality, with 19.98% and 20.34% of its area under high and very high susceptibility classes, respectively. For the analysis of building exposure to co-seismic landslide susceptibility, a building footprint map is developed and overlaid on the co-seismic landslide susceptibility map. The results show that the Sindhupalchowk and Dhading districts have the largest and smallest number of houses exposed to co-seismic landslide susceptibility. Additionally, when conducting a risk analysis based on susceptibility mapping, as well as considering socio-economic and structural vulnerability in Barhabise municipality, revealed that only 106 (1.1%) of the total 9591 households, were found to be at high risk. As this is the first study of co-seismic landslide risk study carried out in Nepal and covers a regional to the municipal level, this can be a reference for future studies in Nepal and other parts of the world and can be helpful in planning development activities for government bodies.

## Introduction

A landslide is an outward and downward movement of materials in a slope^1^. Among the many natural hazards that occur in mountainous areas throughout the world, landslides are one of the most severe and devastating hazards^2^. Many different intrinsic and extrinsic factors cause or trigger landslide occurrences^3–5^. The intrinsic factors include the geomechanical, geomorphological and hydraulic properties of slope materials, whereas the extrinsic factors include factors such as rainfall and earthquakes. In many cases, anthropogenic activities, including nonengineered excavation in hillslopes, influence the occurrence of landslides^5–8^.

Nepal, being in the central part of the young Himalayas, experiences extreme geological and geotechnical problems, including mass wasting geohazards such as landslides^9,10^. Most of the landslides in Nepal occur in the monsoon season and are triggered by heavy rainfall^11,12^. According to the Ministry of Home Affairs (MOHA) 2022^13^, between 2015 and 2022, approximately 2300 landslide events occurred in Nepal that claimed dozens of lives and property worth millions of dollars, but there were other plethora of minor landslide events that are generally not recorded by the government body. On the other hand, more than 25,000 landslides were triggered by the 2015 Gorkha earthquake (7.8 Mw), and its aftershock in central Nepal^14^ infers the importance of co-seismic landslide studies in reducing disaster risk. The development of a proper susceptibility map considering effective causative factors and their use in the analysis and design of mitigation help to minimize the loss from landslides^8^. Many studies have been carried out on landslide susceptibility mapping along the Himalayas, but they are limited to a small spatial area and do not consider the direct influence of an earthquake^4,15^. The complex mechanism of landslide occurrence due to earthquakes is not well understood, and there are very limited studies that have considered landslide susceptibility analysis considering earthquakes.

Different approaches have been implemented for landslide susceptibility mapping, such as different kinds of statistical methods. Models like weight of evidence and frequency ratio are bivariate statistical methods and logistic regression is a multivariate statistical method. Since the past decade, artificial intelligence (AI) methods for instance, machine learning (ML) methods for example: artificial neural network (ANN), support vector machine (SVM), random forest (RM), extremely randomized trees (ERT), extreme gradient boosting (XGB), and classification and regression trees (CART) have become popular methods for landslide susceptibility mapping^16,17^. In the conventional method of landslide susceptibility modeling, including the heuristic approach and the knowledge-based approach, a high level of expertise is needed along with a strong knowledge of geomechanical and geomorphological factors influencing the occurrences of landslides. The methods are cumbersome to apply. Similarly, physical-based modeling also needs more time to compute the relation between landslides and different parameters of slope-forming materials. These limitations can be mitigated by applying ML models for susceptibility mapping^18^. Due to the complex relationships between landslide conditioning factors and landslide susceptibility, ML methods are superior to conventional approaches in these domains^17,19^. Since ML algorithms are robust, can handle missing values, and can optimize the fitting of a model, i.e., avoid overfitting, ensemble ML techniques such as RF, XGB, ERT, and CART are widely used in the field of data classification and regression^20,21^.

Some studies are carried out in different part of Nepal using different statistical methods^22,23^ like index entropy, certainty factor method, frequency ratio etc. A few machine learning methods are applied for landslide susceptibility mapping in watershed level study in some districts^24–28^. In addition to being suitable for large areas, they do not require that the environmental factors be distributed normally. Pyakurel et al.^21^ performed landslide susceptibility mapping in regional level employing different ML models, and concluded that ERT model has best prediction capability among the models employed. Moreover, ERT remove the discretization threshold values through optimization for node splitting which makes it robust and more accurate^29,30^. Similarly, other studies have reported that performance of ERT is better than other ML models form their studies for flood and landslide susceptibility mapping^29,31^ Therefore, ERT model is applied for the susceptibility mapping of co-seismic landslides distributed in a large area.

A co-seismic landslide susceptibility map, when considered from the perspective of disaster risk reduction and management, can play a significant role in associated risk reduction and management by providing information on the likelihood of landslide occurrence. When landslide-prone locations are well recognized, it is simple to develop risk reduction and management plans. By utilizing the different methods to determine the nature and extent of hazard, exposure and vulnerability any associated risk can be assessed^32^. Different studies were carried out using quantitative, qualitative and semi-quantitative risk assessment methods^33–37^. Semi-quantitative method was used by Althuwaynee et al.^37^ where vulnerability was assumed to be constant and the product of hazard probability and exposure was considered landslide risk. Bell et al.^36^ used quantitative risk assessment using all three components, i.e., hazard, vulnerability and exposure, to prepare risk map. By combining the probability of hazard and vulnerability, landslide risk map was prepared^38^.

Buildings in the higher risk class are exposed to higher risk. While the buildings in lower risk class have minimal risk. This crucial information on the exposure of buildings to disasters is important for developing resilient communities and useful in disaster risk reduction of the community. Therefore, this study focuses on preparing a susceptibility map on regional and municipal scales with building exposure analysis and risk map for a municipal level to highlight the importance of co-seismic landslide risk study.

### Study area

**Figure 1:**
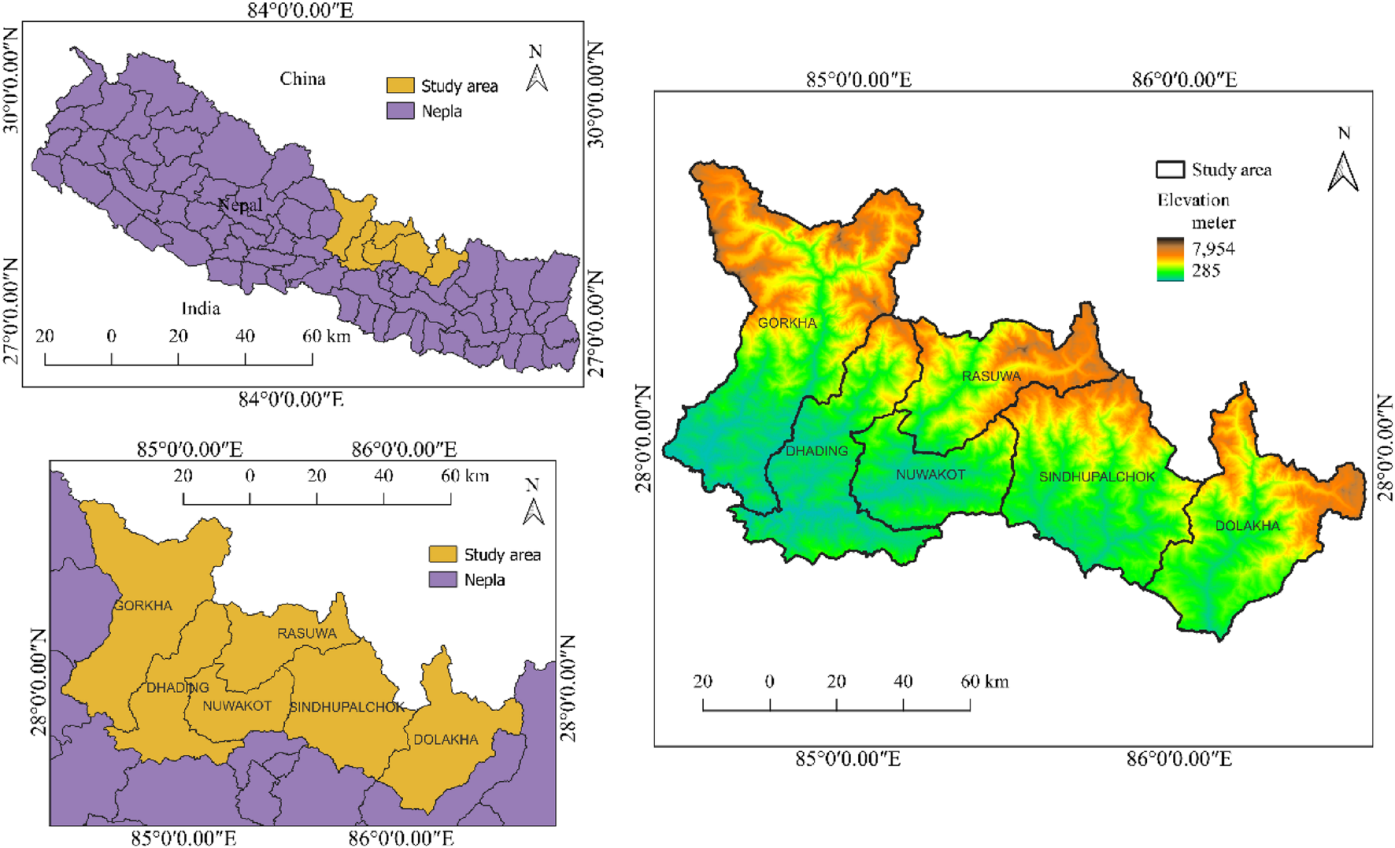
Study area.

With a total area of 12,886.95 km^2^, the research area is located in Nepal’s central region between 27.47° N to 28.75° N and 84.42° E to 86.55° E. It belongs to both lesser Himalaya and higher Himalaya geological zones. The Himal Group formation is the main geological formation in the higher altitude area, and it contains highly graded metamorphic rocks such as gneisses, kyanite quartzites, and thin layers of marble. The Ranimatta Formation, which consists of grayish greenish gray phyllites gravelstones with conglomarates and white massice quartzites in the upper part, is the dominant geological unit in the lesser Himalaya. The geomorphology of the study area has been influenced by tectonic activity, weathering, erosion, and slope failures, resulting in steep peaks and deep gorges. The slope of the study area ranges from 0° to 87.98°, with significant elevation changes between 285 and 7954 m. Intense rainfall, dense populations, and earthquakes can all increase the risk of fatal landslides in the area considered^14^. The study area is shown in Fig. 1.

## Methodology

**Figure 2:**
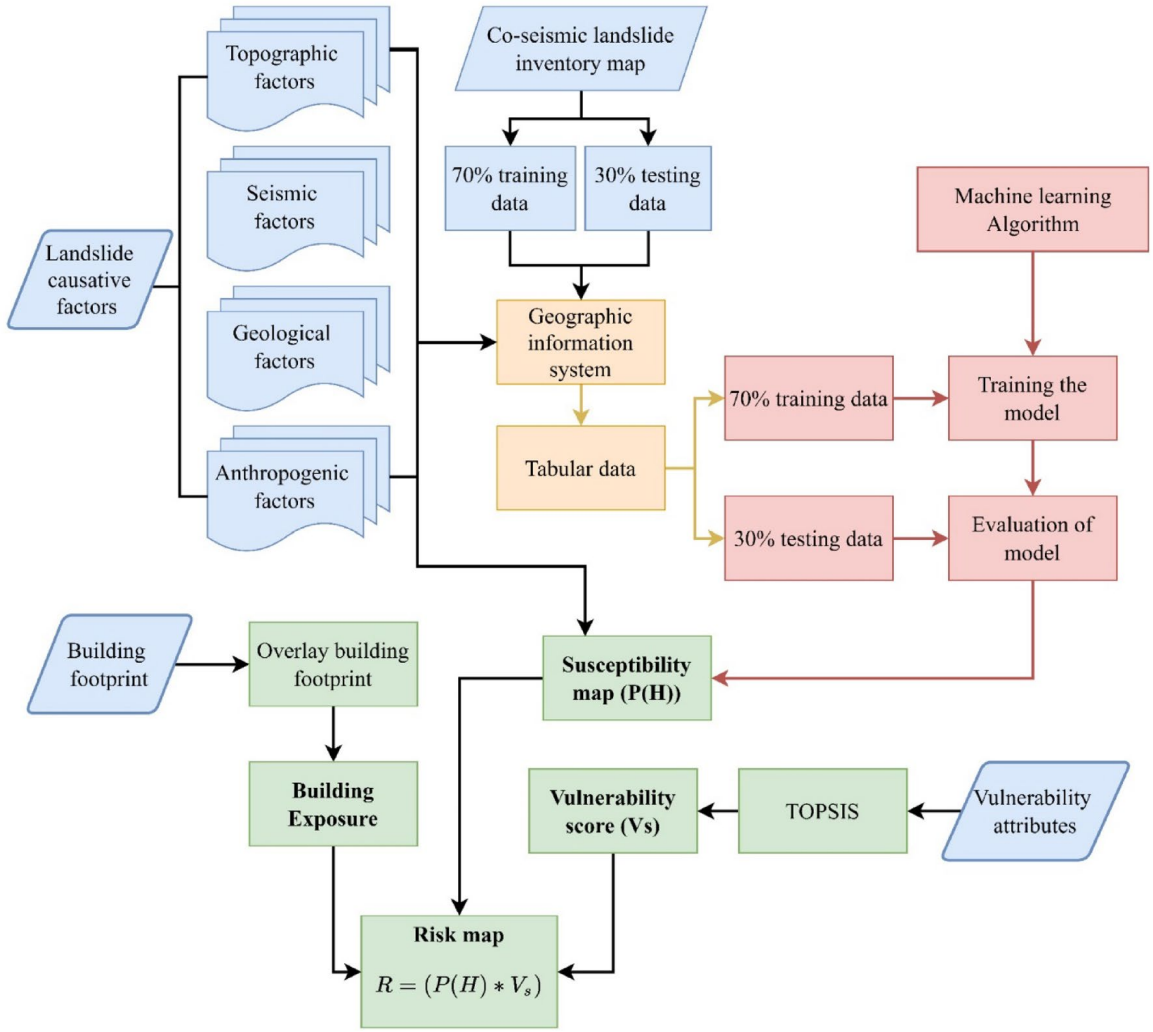
Research framework.

The present study analyzed an approach for the mitigation of co-seismic landslide hazards, considering the 23,164 landslides that occurred after the 2015 Gorkha earthquake (7.8 Mw) in Nepal. The distribution of these landslides in relation to the rupture plane of the earthquake is analyzed and is considered one of the major factors for landslide occurrence. Peak ground acceleration is another important factor considered for the analysis of these co-seismic landslides. Nine other factors, including slope, aspect, total curvature, topographic ruggedness index (TRI), distance to rivers, distance to roads, land cover and land use, and geology, are also considered contributing factors for landslide occurrence. Quantum Geographic Information System (QGIS)^39^ is used for all geospatial data preparation and calculation. Moreover, one of the machine learning methods, i.e., the extreme randomness tree (ERT), is used for landslide susceptibility mapping, and the results thus obtained are validated by plotting the success rating curves. A building footprint map is created using data from the open street map (OSM), which is then overlaid on the susceptibility map, and zonal statistics for the building footprint are calculated using QGIS. After analyzing the zonal statistics of the buildings, the exposure of the buildings in each susceptibility class is evaluated. Finally, socio-economic and structural vulnerability assessment is carried out using “Technique for Order Preference by Similarity to an Ideal Solution” (TOPSIS) method. To evaluate the landslide risk, the product of probability of landslide and the vulnerability is used. The overall research framework is given in Fig. 2.

### Data acquisition and processing

**Figure 3:**
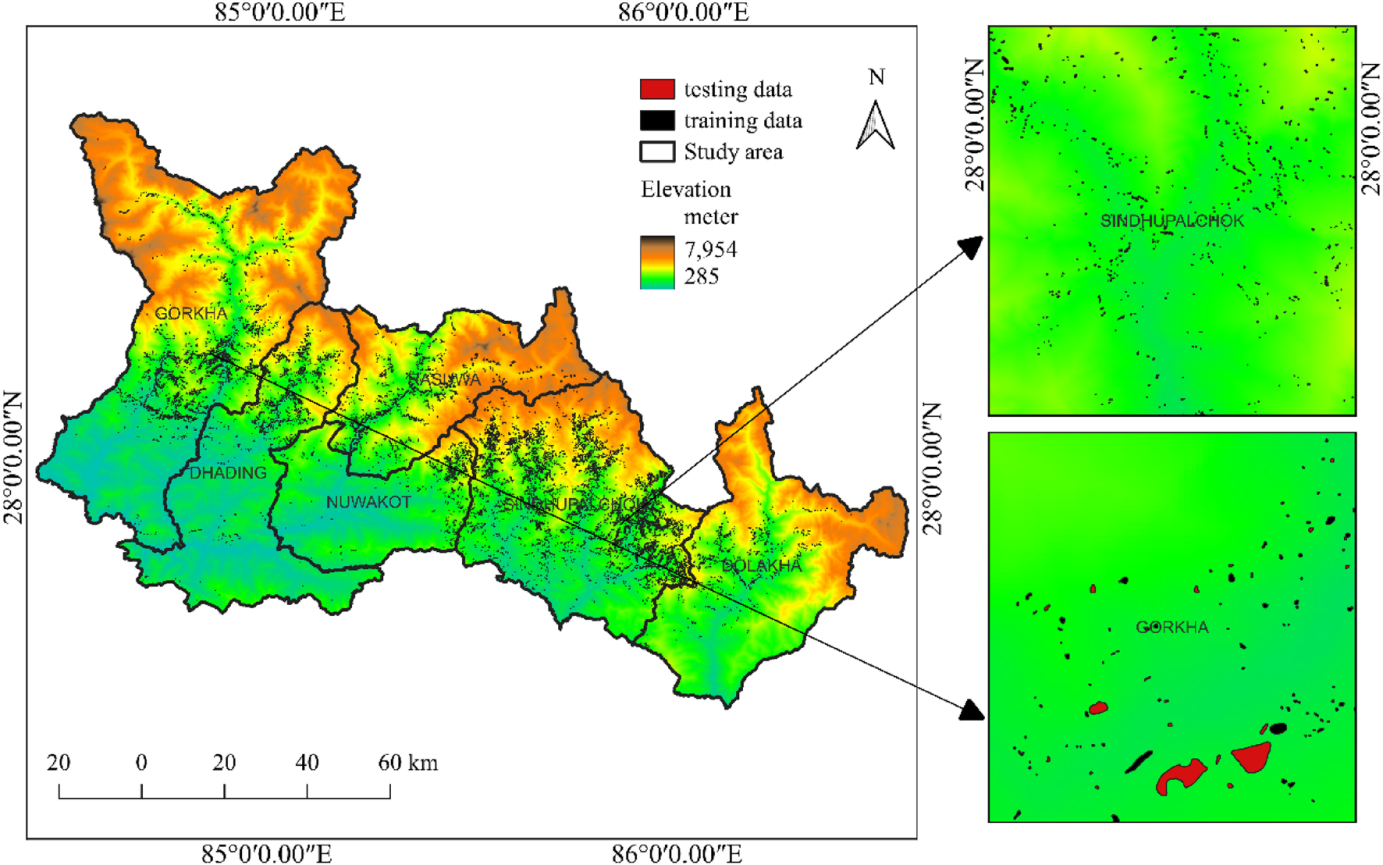
Landslide inventory map with training and validation data sets.

The landslide inventory map containing 23,164 post earthquake landslide polygons was prepared the study of Roback et al.^14^. Each landslide was represented in the analysis by a point at its centroid. Similarly, to consider the non-landslide samples, random points were generated within the study boundary and the points inside landslide polygons were carefully removed. To overcome the imbalance in training and testing around 23,000 points were generated for non-landslide data. The training-to-validation data ratio in most research ranges from 60:40 to 70:30^40,41^. In this study, the ratio is kept at 70:30, which means that out of 23,164 landslides, 16,214 are considered the training set and the rest the validation set. Figure 3 depicts the landslide inventory map with training and validation sets. The study area’s digital elevation model (DEM) was obtained from the US Geological Survey (USGS). The downloaded DEM had a grid size of 30 m by 30 m. The slope, aspect, total curvature, topographic ruggedness index (TRI) and distance to rivers were derived using the DEM. Similarly, the layers of distance to faults and geology were created using data from the Department of Mines and Geology (DMG). From USGS, the layer peak ground acceleration (PGA) was obtained. Based on the research of Elliott et al.^40,41^, the layer distance to the rupture plane was calculated. Furthermore, the land cover and land use layer was produced using Sentinel-2 satellite remote sensing data, while the distance to the road layer was produced using International Centre for Integrated Mountain Development (ICIMOD) data. All data collected from various sources are processed using GIS.

### Causative factors

The occurrence of landslides is influenced by a wide range of causative factors. Seismic parameters such as ground acceleration, distance from the rupture plane, and other topographic, geological, and hydrological factors all contribute to the occurrence of landslides. Twelve causative factors are taken as major factors contributing to the occurrence of landslides as considered in the previous study^21^. Information about these factors is given in Table 1.

**Table 1:** Co-seismic landslide causative factors for this study.

S. n	Landslide causative factors	Resolution	Data source
1	Elevation (DEM)	30 m × 30 m	USGS (DEM) (EarthExplorer (usgs.gov))
2	Aspect	30 m × 30 m	Derived from USGS (DEM)
3	Slope	30 m × 30 m	Derived from USGS (DEM)
4	Total curvature	30 m × 30 m	Derived from USGS (DEM)
5	Topographic ruggedness index (TRI)	30 m × 30 m	Derived from USGS (DEM)
6	Distance to rivers	30 m × 30 m	Derived from USGS (DEM)
7	Distance to faults	30 m × 30 m	DMG (Department of Mines and Geology—Home-page (dmgnepal.gov.np))
8	Distance to rupture	30 m × 30 m	Elliott et al.^42^
9	Peak ground acceleration	30 m × 30 m	USGS shake map
10	Geology	30 m × 30 m	DMG (Department of Mines and Geology—Home-page (dmgnepal.gov.np))
11	Distance to roads	30 m × 30 m	ICIMOD (ICIMOD|RDS)
12	Land use land cover	10 m × 10 m	ESRI Global Landcover (Sentinel-2 10m Land Use/Land Cover Timeseries Downloader (arcgis.com))

### Topographic factors

**Figure 4:**
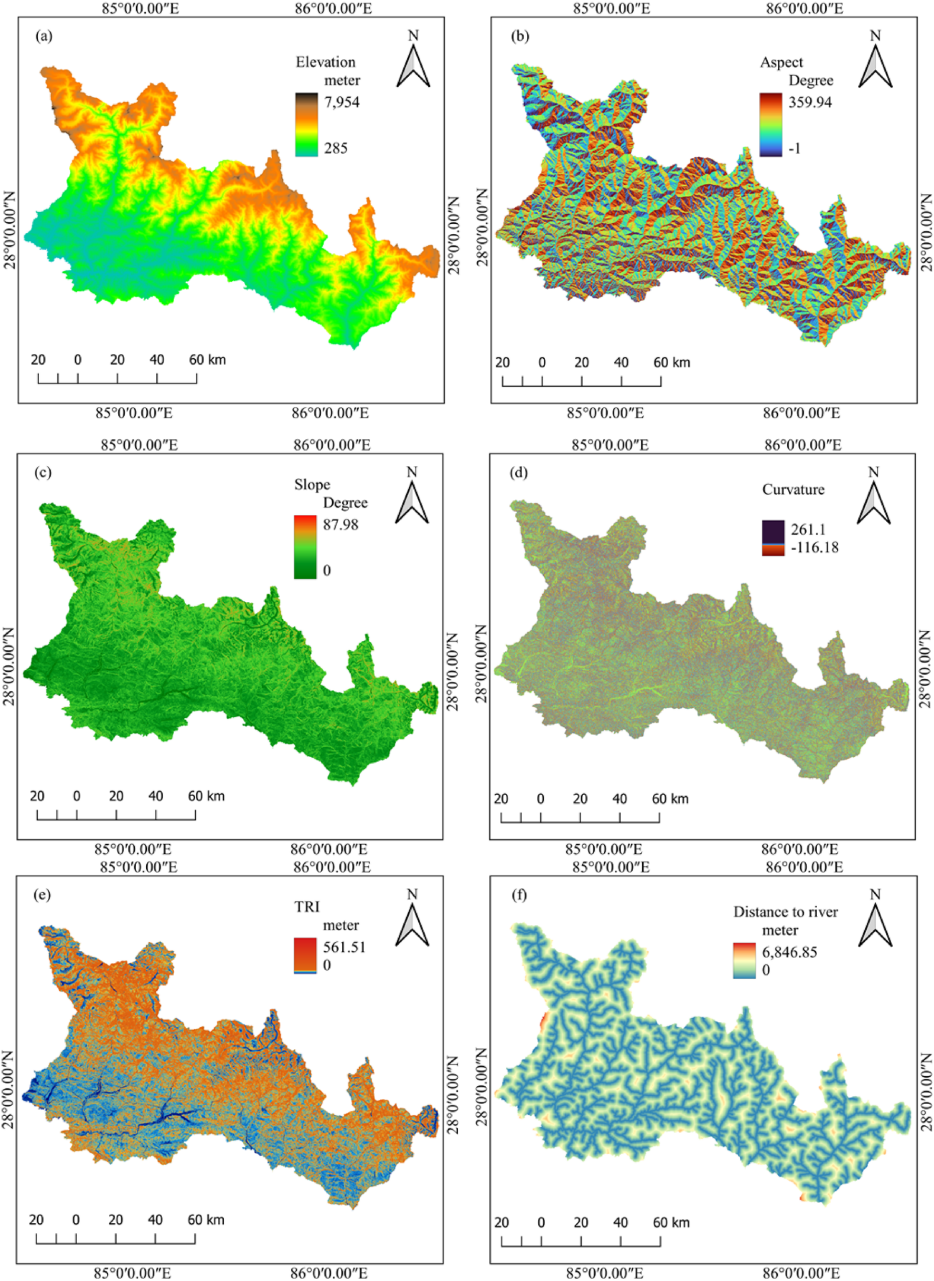
Topographical landslide causative factors: (**a**) elevation, (**b**) aspect, (**c**) slope, (**d**) curvature, (**e**) TRI, and (**f**) distance to river.

Under topographic factors, elevation, aspect, slope, total curvature, topographic ruggedness index and distance to rivers are considered. The factor map of topographic factors is shown in Fig. 4.

#### Elevation

Elevation is one of the major factors controlling the rainfall distribution, vegetation cover, ground water and weathering of rocks^43^. These factors directly or indirectly influence the occurrence of landslides. The elevation also plays a role in the occurrence of post-earthquake landslides^14^. Therefore, elevation is considered one of the major causative factors in this study.

#### Aspect

The aspect is one of the important factors for the occurrence of landslides, as it can influence moisture, precipitation, and solar radiation^2,44,45^ which play an important role in groundwater and rock strength, which in turn can play an important role in the occurrence of landslides. The slope aspect and the direction of seismic acceleration may have some association. Therefore, aspect is considered one of the main contributing factors to landslide occurrence.

#### Slope

Instability arises on slopes due to the self-weight of materials triggered by rainfall and strong ground motion^8,46^; therefore, susceptibility is directly connected with the surrounding slopes; hence, the slope is one of the critical factors for assessing susceptibility^4,17^. The slope angle in the study area ranges from 0 to 87.98° in the study area considered.

#### Total curvature

Another factor to consider is curvature, which represents the maximum slope direction. According to Pradhan et al.^47^, concavity is a negative curvature, flatness is a zero curvature, and convexity is a positive curvature. Flow and slide are more likely in flatness than in convex curvature. The total curvature in the study area ranges from − 17.56 to 54.23.

#### Topographic ruggedness index

The topographic ruggedness index (TRI) measures the elevation difference between adjacent DEM cells. TRI is a morphometric measure that describes the variety of a land surface; it classifies the terrain as smooth or rugged^48^.

#### Distance to rivers

The distance to the river is the stream proximity from the landslides to the nearest natural drainage. Natural drainages can adversely affect the stability of slopes by toe incisions and by saturating slope-forming materials due to an increase in water level^49,50^. In general, the closer the distance from the river is, the higher the density of the landslide^4,25^.

### Seismic factors

**Figure 5:**
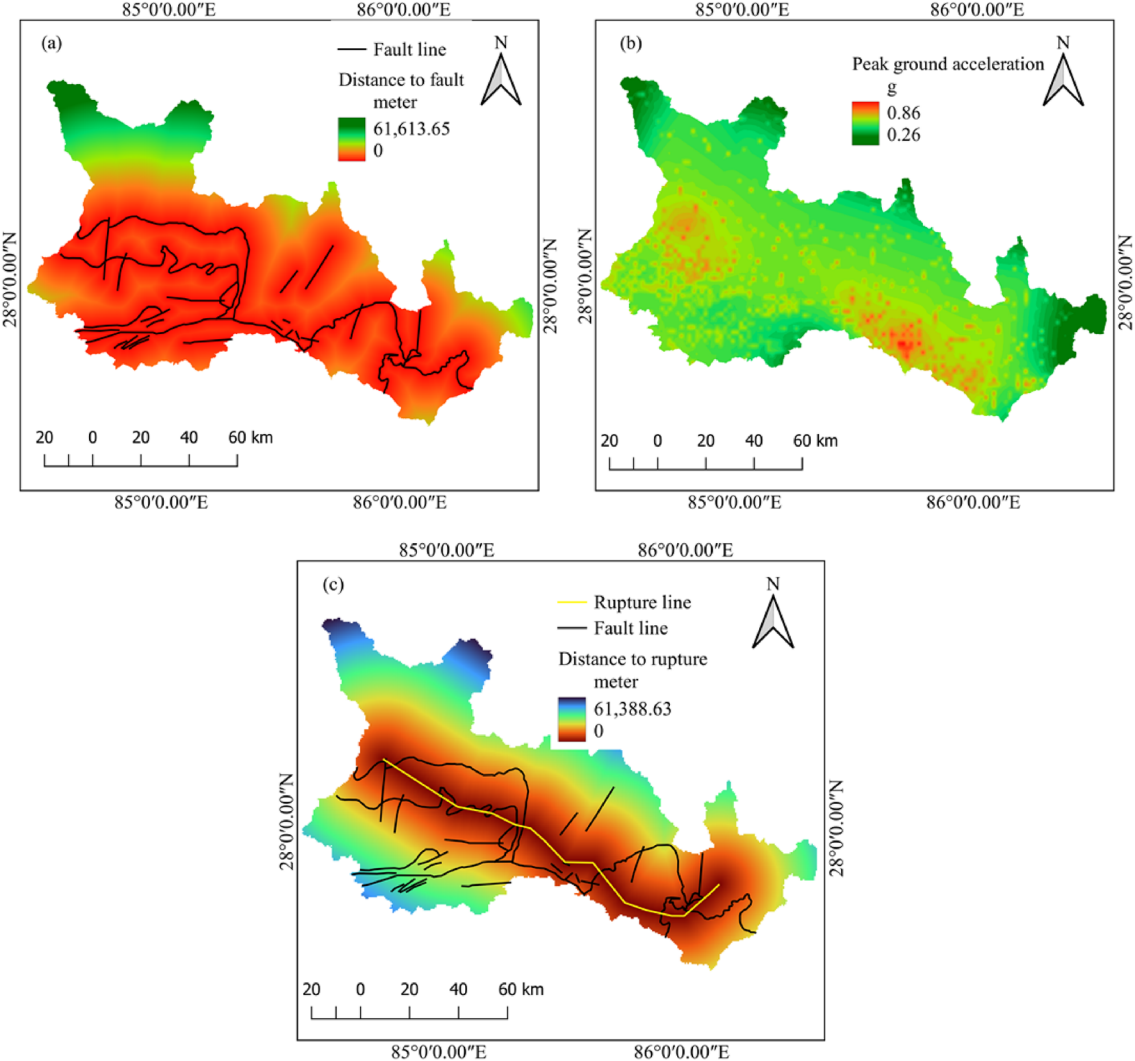
Seismic landslide-causing factors: (**a**) distance to fault, (**b**) peak ground acceleration, and (**c**) distance to rupture plane.

Under seismic factors, distance to faults, peak ground acceleration and distance to rupture plane are considered. The factor map of seismic factors is shown in Fig. 5.

#### Distance to faults

Faults are discontinuities in the rock mass that are generated by tectonic or seismic activities. The areas around the fault are more prone to landslides as the rocks in the proximity are disturbed and are generally weak. Faults also play a role in the movement of ground water, playing an important role in landslide occurrences^7^. During an earthquake, a fault may act as a barrier for the propagation of seismic waves.

#### Peak ground acceleration (PGA)

Earthquake characteristics, such as magnitude and depth, play an essential role in co-seismic landslide distribution^51,52^. With the same magnitude of the earthquake, the effect can differ based on the peak ground acceleration and amplification factors^53^. Generally, higher destruction and more landslides are likely to occur with the increase in magnitude of an earthquake and the peak ground acceleration^54^. Hence, peak ground acceleration (PGA) is considered one of the thematic layers for susceptibility analysis in this study.

#### Distance to the rupture plane

Co-seismic landslides and distance from the rupture plane have a strong positive correlation; in most cases, as distance from the rupture plane increases, the frequency of landslides decreases^55^. A denser distribution of co-seismic landslides closer to the rupture plane and a sparse distribution away from the rupture plane were also reported by^56^. Using remote sensing techniques, for instance, interferometric synthetic aperture radar (InSAR) data and synthetic aperture radar (SAR) interferometric analysis, some studies have estimated the rupture plane for the Gorkha earthquake^57,58^. In this study, the rupture plane is determined based on the study by Elliott et al.^42^.

### Anthropogenic factors

**Figure 6:**
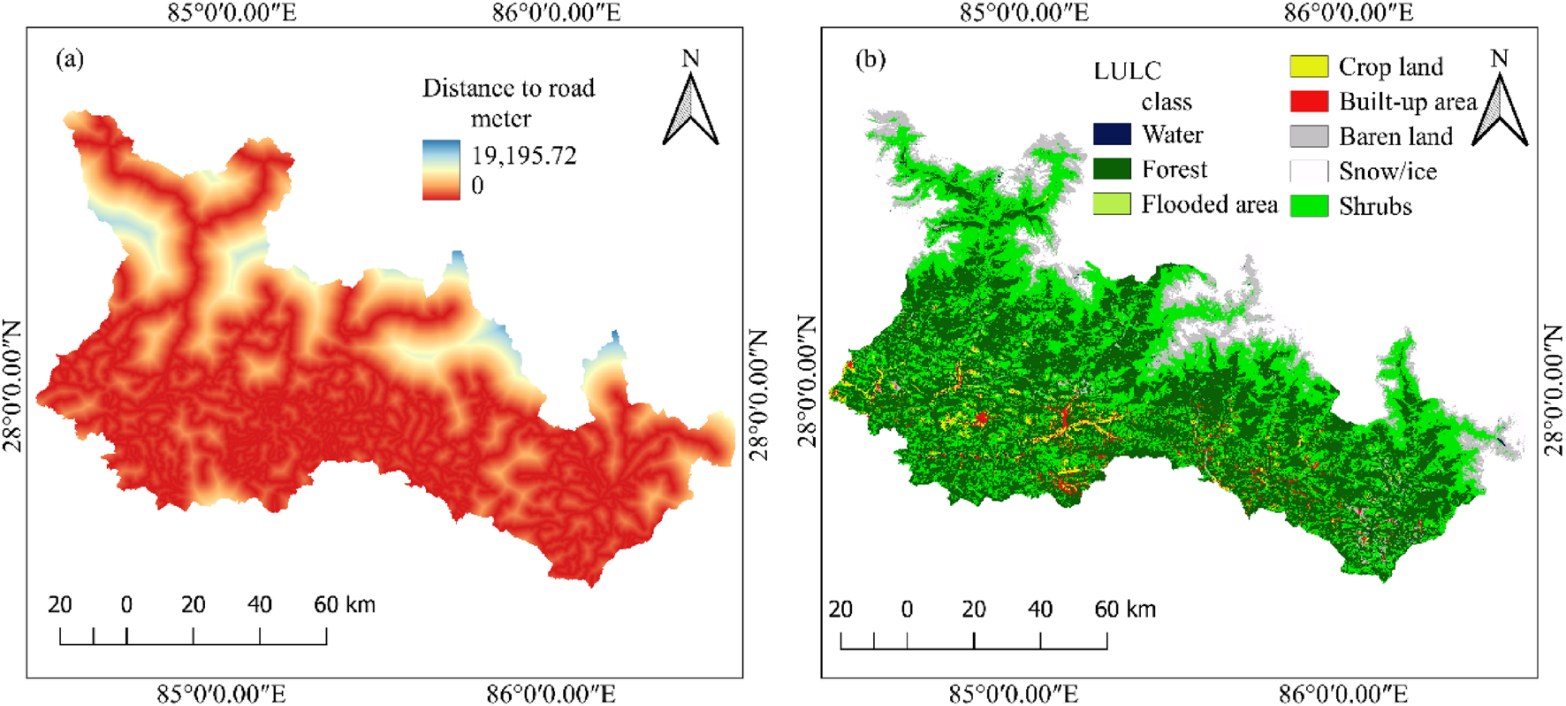
Anthropogenic landslide causative factors (**a**) distance from the road, (**b**) land use and land cover.

Under anthropogenic factors, distance to roads, land use and land cover are considered. The factor map of anthropogenic factors is given in Fig. 6.

#### Distance to roads

The stability of slopes is directly affected by anthropogenic factors such as road excavations^59^. Due to unplanned and nonengineered road construction, the mountainous regions of Nepal have experienced many landslides^7^. A significant number of landslide occurrences around roads after the 2015 Gorkha earthquake were reported by Mcadoo et al.^60^. Therefore, the factor distance to road is considered an important factor for landslide occurrence.

#### Land use and land cover (LULC)

Landslide occurrence is greatly influenced by LULC; for example, infrastructure development activities such as road excavation and urbanization have an impact on landslide occurrence^45,60^. The LULC factor is classified into seven classes: water bodies, forest, shrubs, cultivated land, barren land, snow/ice cover and built-up area. The spatial resolution of LULC used in this study is 10 m × 10 m^61,62^.

### Geology

**Figure 7:**
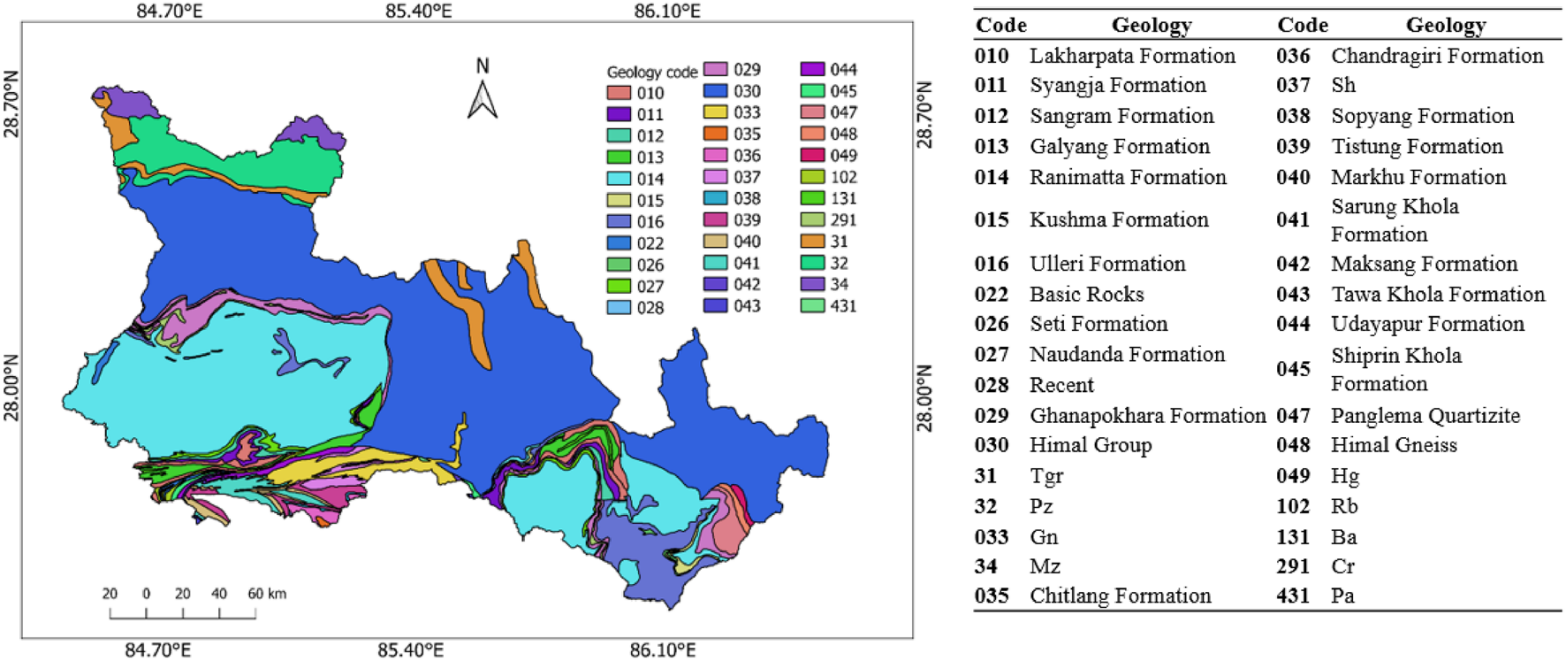
Geological setting of the study area.

Geological formation and setting affect the amplification and propagation of the ground motion of seismic waves because this geology plays a significant role in the co-seismic landslide distribution. The Himalayas in Nepal are fragile and have very intricate geology with steep slopes that are prone to landslides during strong ground motion. The details on the geology of the study area are given in Fig. 7.

### Building the footprint map

**Figure 8:**
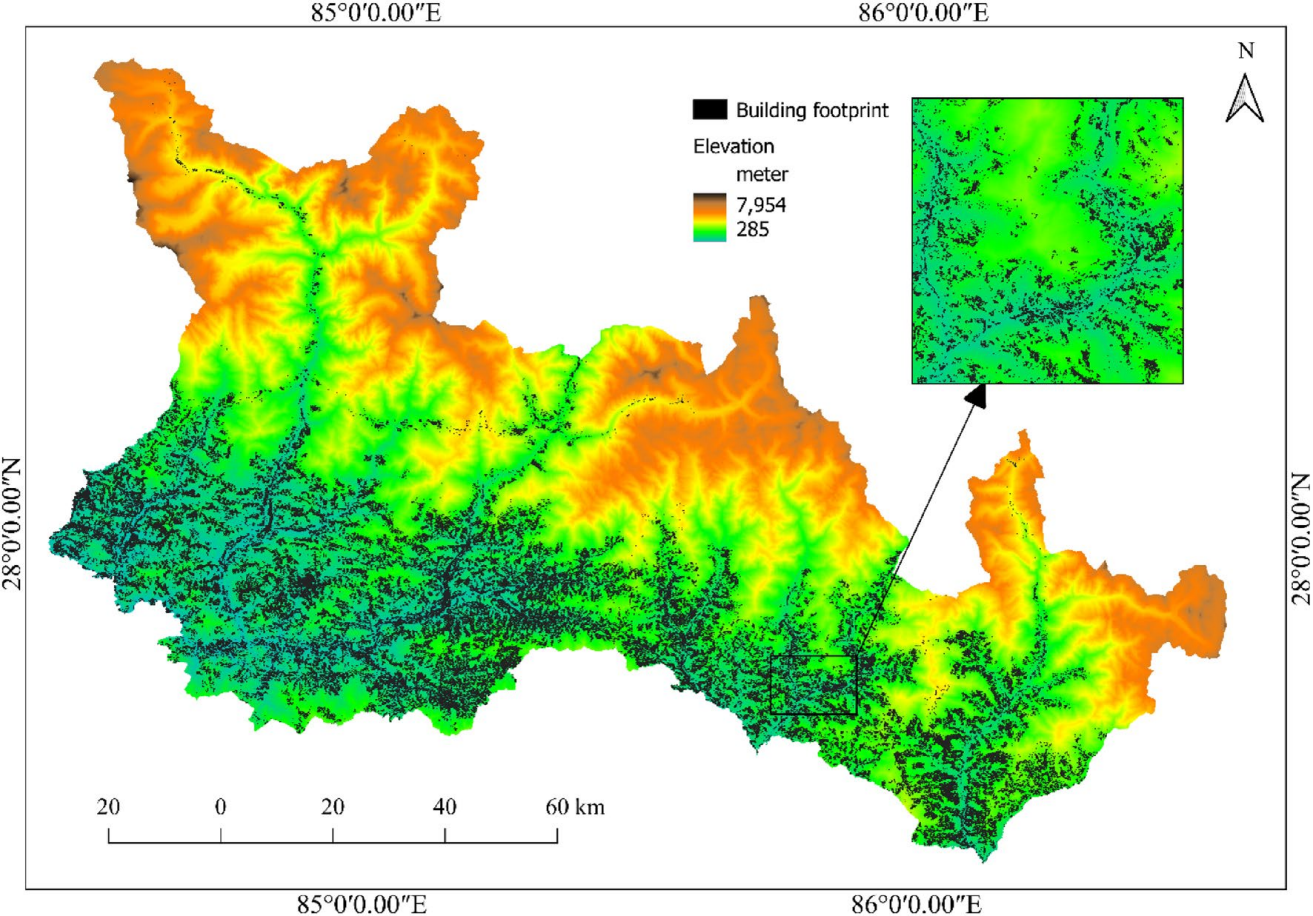
Building footprint map over the study area [source: open street map (OSM)].

To investigate the buildings’ exposure to co-seismic landslides, it is necessary to prepare a building distribution map over the study area and overlay it on the co-seismic landslide susceptibility map. The building footprint data over the study area are taken from open street map (OSM). A total of 477,798 buildings are observed in the study area based on OSM data. The buildings are primarily concentrated at lower elevations and along the river basins. To examine the exposure of buildings, the building footprint map (Fig. 8) thus developed is overlayed on the co-seismic landslide susceptibility map. The districts and local units with the highest building exposure to high co-seismic landslide susceptible areas are further analyzed.

### Modeling of landslide susceptibility

Modeling of landslide susceptibility is carried out using a machine learning (ML) approach. It is a kind of artificial intelligence (AI) that is used for data analysis, classification, and regression problems. There are two types of ML generally used in data classification problems, i.e., supervised and unsupervised models. By analyzing past data, supervised ML models learn the patterns and relations between the target and feature values and then predict the target pattern based on the input features. In this study, an extremely randomized tree (ERT) model is used for the modeling of co-seismic landslide susceptibility. The extremely randomized tree (ERT) model is the extended version of the random forest (RF) model, which is also known as the extra tree model. It is a well-structured supervised machine learning model that is used for regression and classification of data sets. The ERT model generates more reliable results than the RF model by using all sample data sets during the training process, and on the basis of decision tree learning, ERT is a more advanced model that contains numerous decision trees^63^. The ERT model has three major advantages. (1) This model has better accuracy, as the results are determined by the maximum number of votes. (2) It is efficient in handling a large number of multidimensional input data sets. (3) It can also rank features during algorithm implementation by variable importance.

**Figure 9:**
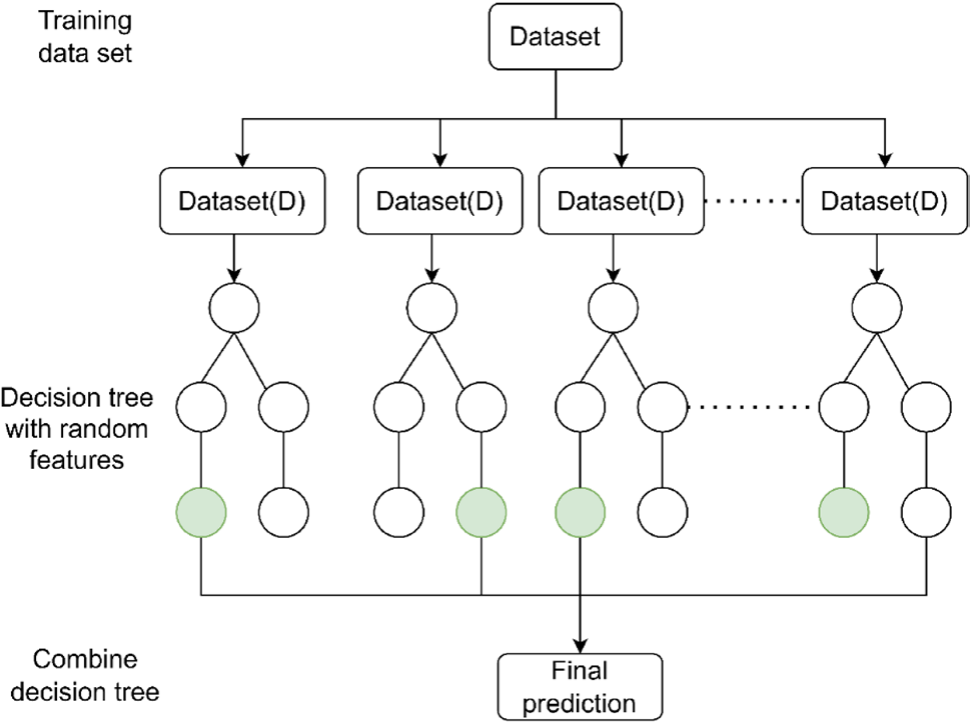
Flow chart of extremely randomized tree model.

The ERT algorithm was proposed by Geurts et al.^30^. It is similar to random forest algorithm, but it has two major differences. The first considers whole data into the construction of one decision tree with random features sets. The second is that it randomly splits the nodes with features forming a large number of trees. ERT’s larger tree size and numerous splits aim to reduce bias, enhancing its generalization performance (Fig. 9). The process of ERT splitting comprises three parameters: the attribute selection strength parameter (K), the minimum number of samples needed to split a node (nmin), which defines the average output amount, and the number of trees (M), which dictates the extent of variance reduction^64^. ERT stands out for its robustness against overfitting, strong resistance to noise, and adeptness in managing high-dimensional datasets without pre-selecting features^64^.

In this paper, the ERT model is applied by using the scikitlearn.ensemble library in Python. The extremely randomized tree classifier model in the scikitlearn.ensemble library is used to quantitatively measure the contribution of each causative factor.

## Vulnerability assessment

Vulnerability assessment is a crucial part in understanding the risk and mitigate it. To understand the risk associated with hazard event, event occurrence probability, exposed element and vulnerability need to be quantified^65^. Risk associated with natural hazard can be defined as the function of hazard, exposure, and vulnerability^66^ as shown in Eq. (1)

1 $$R=f(H,E,V)$$

where H = hazard, E = exposure, V = vulnerability.

In this study, vulnerability and risk assessment was carried out at local level i.e., ward level. The ward level data was collected from national population and housing census 2021^67^. For vulnerability calculation, the structural properties of buildings like outer wall materials, foundation types are considered as a physical parameter. Similarly, for the socio-economic variables, economically active age group from 15 to 59 and economically inactive age group 0–14 and greater than 65 are considered. Female to male ratio and female population are also considered under the socio-economic parameters. Literacy affects the people’s reach on disaster information and risk understanding, therefore the literacy parameters are considered as well.

For the total vulnerability score calculation TOPSIS is used. TOPSIS is an improved method for vulnerability score calculation based on the entropy weight; the positive ideal solution and negative ideal solution. Based on the distance from either positive and negative ideal solution, the degree of dispersion is measured.

For TOPSIS analysis, after normalization, entropy weight is calculated. Entropy weight method (EWM) is one of the popular methods in the decision-making process^68^ since it relies on the information gained from each index value and uses inherent law to calculate weight for each attribute and weightage according to variation of index^69^. First attributes were normalized using vector normalization as of their cardinality^70,71^.

Positive cardinality is normalized using Eq. (2)^70^.

2 $${n}_{ij}=\frac{{a}_{ij}}{\sqrt{\left({\sum }_{1=1}^{{\text{j}}={\text{n}}}\left({a}_{ij}^{2}\right)\right)}}$$

Negative cardinality is normalized using Eq. (3)^70^.

3 $${n}_{ij}=1-\frac{{a}_{ij}}{\sqrt{\left({\sum }_{1=1}^{j=n}\left({a}_{ij}^{2}\right)\right)}}$$

where ${a}_{ij}$ represent the original data and $i$ represent the municipality index and $j$ represent the index of attributes. Proportion of each $i$ index for $j$ each attribute was calculated using Eq. (4)^72^.

4 $${p}_{ij}=\frac{{n}_{ij}}{\sum_{j=1}^{n}{n}_{ij}}$$

here ${p}_{ij}$ is known as proportion index whose value in case of zero is replaced by the 0.0001 value to maintain validity for natural log calculation. Thus, obtained ${p}_{ij}$ is then used to calculate entropy (${\varphi }_{i})$ of index $i$ using Eq. (5)^72^.

5 $${\varphi }_{j}=-\lambda \sum_{j=1}^{n}{p}_{ij}{\text{ln}}({p}_{ij})$$

where $\lambda =\frac{1}{{\text{ln}}(m)}$, here m is the number of samples in the data and m = 9.

Weight of entropy for each attribute is defined by Eq. (6)^72^

6 $${{\text{w}}}_{{\text{j}}}=\frac{1-{\varphi }_{j}}{\sum_{j=1}^{m}(1-{\varphi }_{j})}$$

7 $${t}_{ij}=\left[\begin{array}{ccc}{w}_{1}*{p}_{11}& ..& ..\\ {w}_{1}*{p}_{21}& ..& ..\\ ..& ..& {w}_{j}*{p}_{ij}\end{array}\right]$$

Considering ${L}^{+}$ and ${L}^{-}$ are the positive and negative ideal solution, which are the maximum and minimum values in matrix L (${t}_{ij}$) as given in Eqs. (8) and (9)^71^. The positive cardinality is considered for positive solution and negative cardinality is considered for negative ideal solution.

8 $$L^{ + } = \left\{ {\begin{array}{*{20}c} {max\left( {t_{ij} } \right)\;for\;j\; in\; j} \\ {min\left( {t_{ij} } \right)\;for\;j\; in\; j^{ - } } \\ \end{array} } \right.$$

9 $$L^{ - } = \left\{ {\begin{array}{*{20}c} {min\left( {t_{ij} } \right)\;for\;j\;in\;j^{ + } } \\ {max\left( {t_{ij} } \right)\;for\;j\;in\;j^{ - } } \\ \end{array} } \right.$$

here $j^{ + } \;and\;j^{ - }$ are the attributes index with positive and negative cardinality.

The distance from the ideal solution are determined as ${d}_{iw}$ for worst and ${d}_{ib}$ for best scenario as given in Eqs. (10) and (11)^71^.

10 $${d}_{iw}={\sqrt{{\sum }_{j=1}^{n}({t}_{ij}-}{{L}_{wj}}^{-})}^{2}$$

11 $${d}_{ib}={\sqrt{{\sum }_{j=1}^{n}({t}_{ij}-}{{L}_{wj}}^{+})}^{2}$$

For the ranking the distance, weight is calculated as ${I}_{iw}$ as worst-case weight also called relative closeness using Eq. (12)^71,72^.

12 $${I}_{iw}=\frac{{d}_{iw}}{{d}_{iw}+{d}_{ib}}$$

Using this index ($${I}_{iw})$$ for each alternative, different classes or ranks are prepared, and the score is given based on the rank. In this study, worst case is used to calculate weight, which means that highest index represent most vulnerable and lowest index represent the low vulnerability.

## Results and discussions

**Figure 10:**
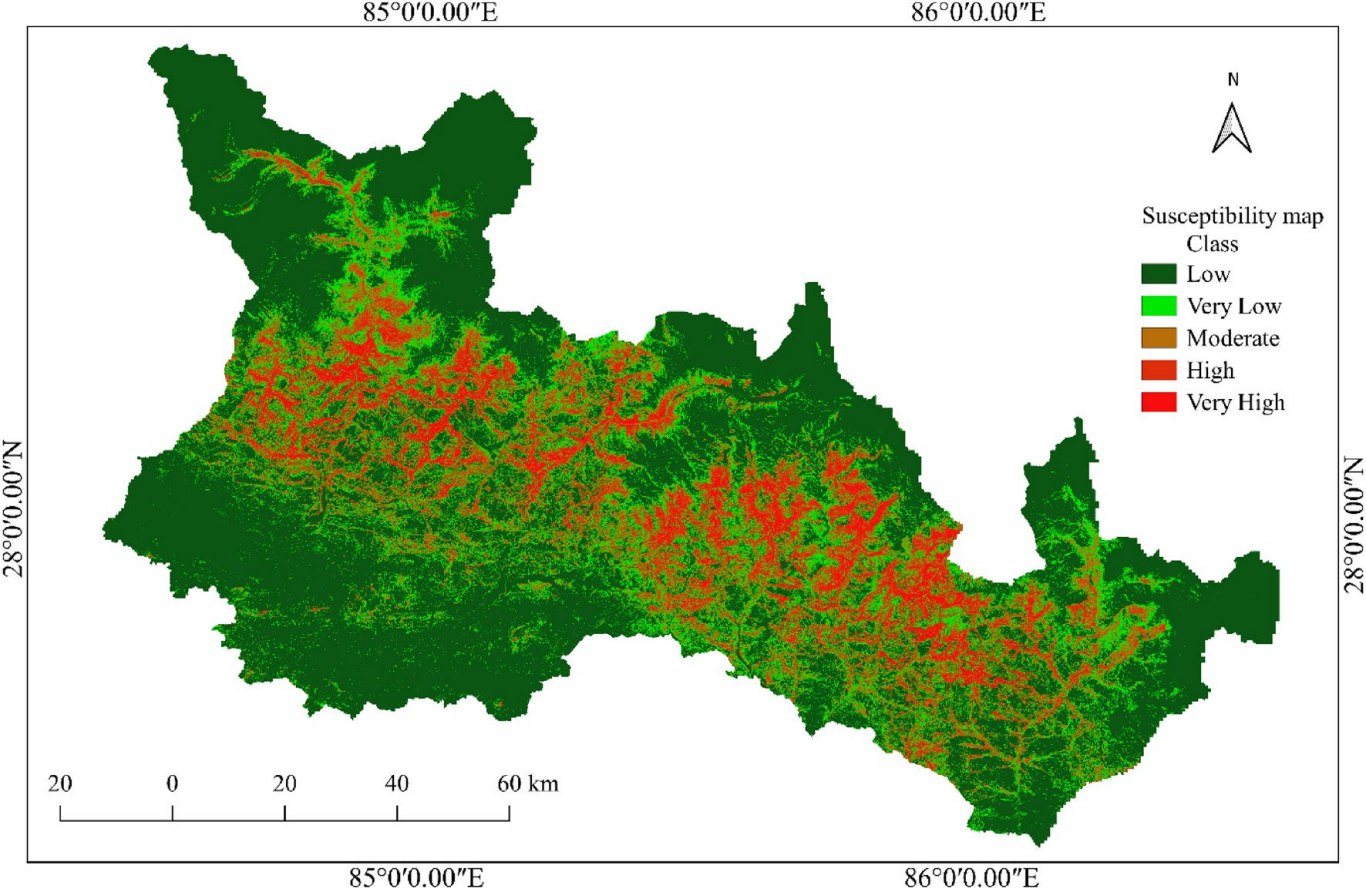
Co-seismic landslide susceptibility map using extra randomized tree (ERT) model.

As shown in Fig. 10, a co-seismic landslide susceptibility map is developed using the twelve input parameters and the ERT ML model. The susceptibility map thus developed is divided into five classes depending on the probability of occurrence of earthquake-induced landslides. The area with a probability of co-seismic landslide occurrence between 0 and 0.2 is classified as a very low susceptibility area. It covers 60.51% of the total study area. This area is considered safe for human activities and infrastructure development.

**Figure 11:**
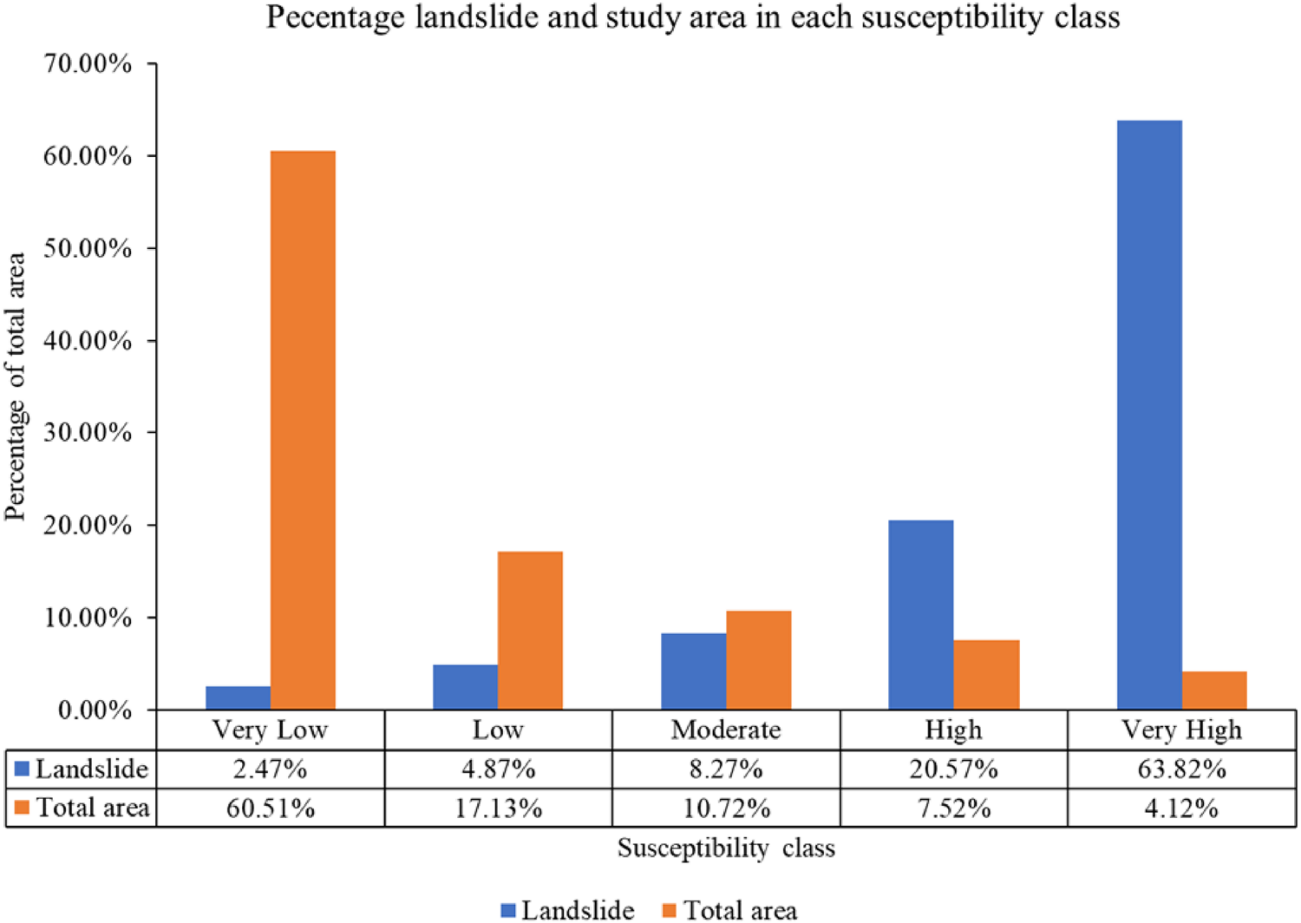
Percentage area and landslides in each susceptibility class in the study area.

The area with a probability of co-seismic landslide occurrence between 0.2 and 0.4 is classified as a low susceptibility area. It covers 17.13% of the total study area. The area with a probability of co-seismic landslide occurrence between 0.4 and 0.6 is classified as a moderately susceptible area. It covers 10.72% of the total study area. Great care should be taken when planning development activities in moderately susceptible areas. Areas with co-seismic landslide occurrence probabilities of 0.6–0.8 are classified as high susceptibility areas, and the remaining areas with co-seismic landslide probabilities of 0.8 to 1 are classified as very high susceptibility areas. A total of 7.52% and 4.12% of the total study area are classified as high and very high susceptibility classes, respectively. It is recommended to avoid these areas for the development of human settlement and infrastructure. The percentage of landslides in each susceptibility class is shown in Fig. 11.

It is observed from the susceptibility map that the high and very high susceptibility areas are in the central part of the study area. Furthermore, these regions are located in the higher part of the mountain, north of the predicted rupture zone. As a result, the slope above and north of the rupture plane is more susceptible to landslides than the slope south of the plane.

### Model validation

The area under the receiver operating characteristic (ROC) curve, the Kappa coefficient, and overall accuracy are often used to compare and evaluate the results obtained from ML algorithms; other statistical approaches, such as root mean square error (RMSE), are also popular statistical indices for the evaluation of model performance^16^. In this study, overall accuracy, ROC curves, the Kappa coefficient, and RMSE are used for the evaluation of model performance. The information on model evaluation is summarized in Table 2.

**Table 2:** Model evaluation.

Model	Accuracy	Kappa	AUC	RMSE
ERT	87.2%	0.744	0.941	0.358

**Figure 12:**
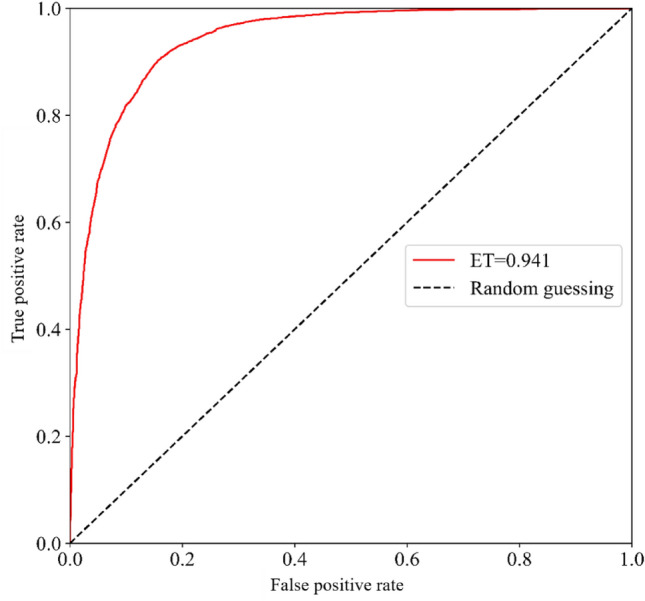
Area under the ROC curve.

Table 2 shows that the overall accuracy of the ERT ML model considering the twelve causative factors, including the three seismic factors, is 87.2%, the kappa coefficient is 0.744, the area under the ROC curve is 0.941 (Fig. 12) and the RMSE value is 0.358. The area under the ROC curve of 0.941 represents excellent model performance. The 0.744 kappa coefficient shows strong model performance with the causative factors considered.

### District wise susceptibility analysis

**Figure 13:**
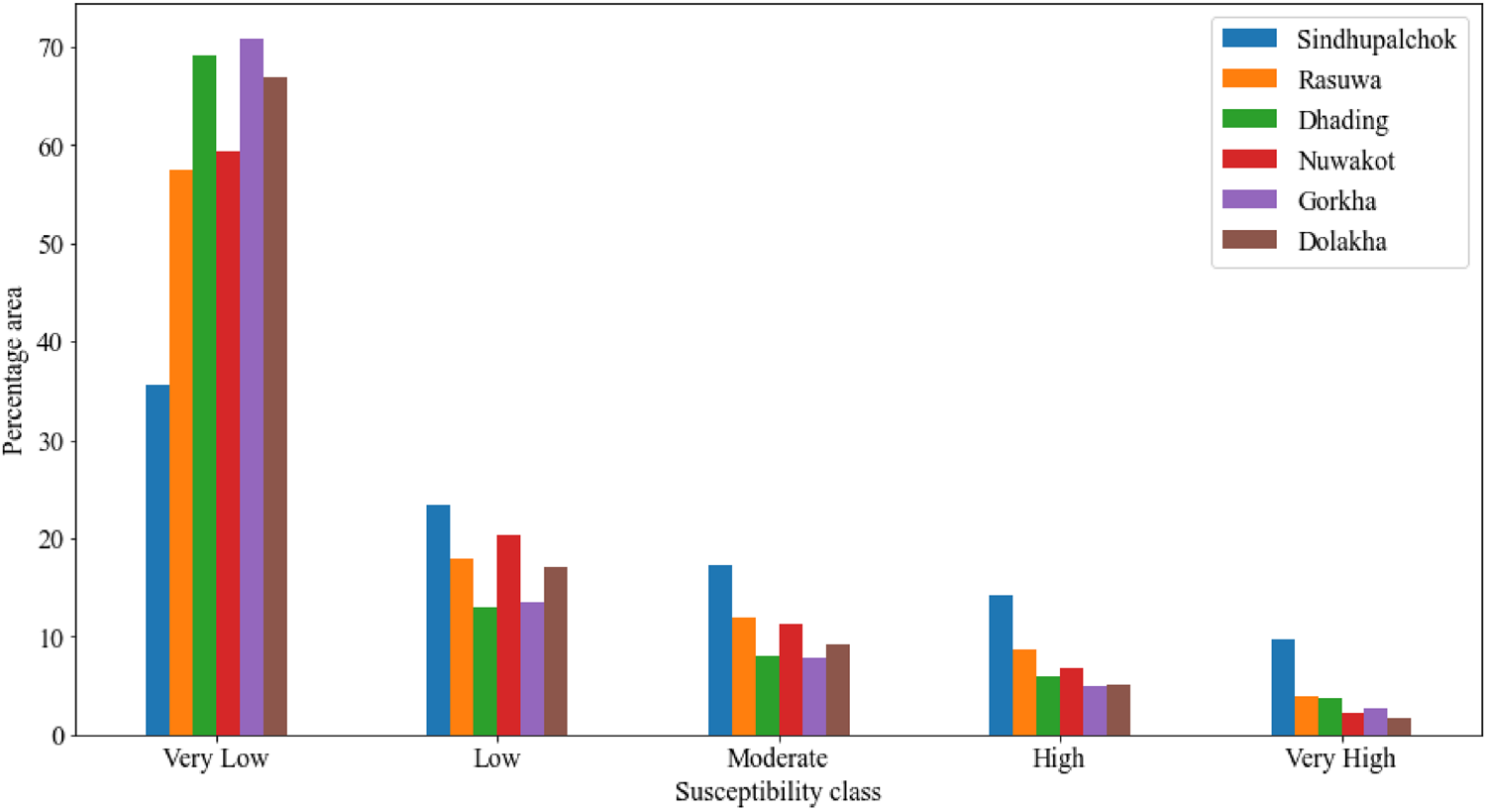
Area under different susceptible classes in different districts.

The study area is very large; therefore, the susceptibility details cannot be observed clearly within the whole susceptibility map; hence, further investigation is carried out district wise as well as for highly susceptible local units. From the district wise analysis, it is observed that the most susceptible district to earthquake-induced landslides is Sindhupalchok. One-fourth of the district falls into the very high or high susceptibility class. Based on the percentage area under the high or very high susceptibility class, Rasuwa, Dhading, Nuwakot, Gorkha and Dolakha followed the Sindhupalchok district. Detailed information is given in Fig. 13. The distribution and the landslide inventory show that the landform above or in the northern part of the surface of the rupture is more susceptible to the landslide.

**Figure 14:**
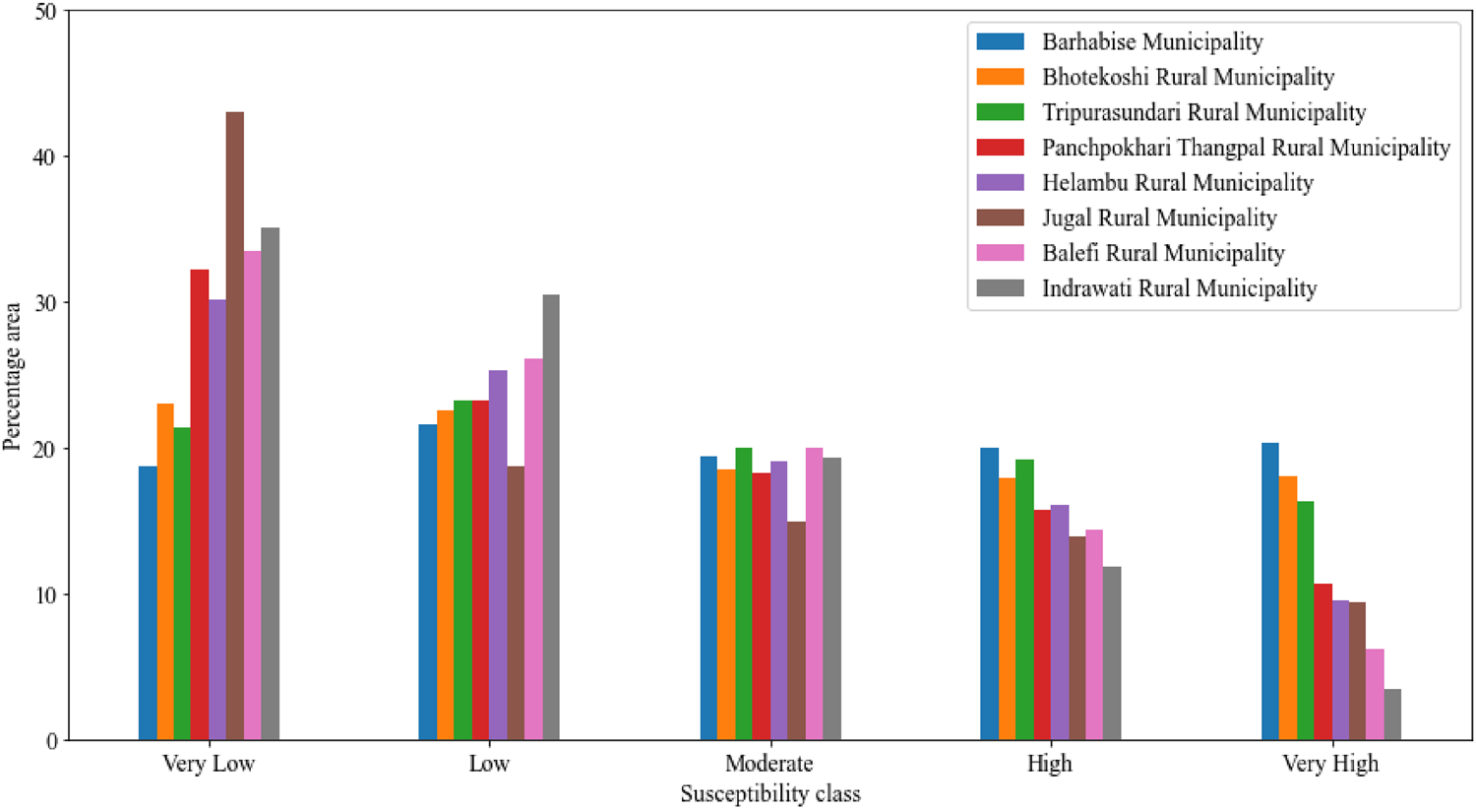
The area under different susceptibility classes in each local unit in Sindhupalchok district.

In Fig. 13, it is identified that the Sindhupalchok district is the most susceptible district. It requires very meticulous analysis and planning for carrying out developmental activities; therefore, further analysis and study are carried out at the local unit level. The area under different susceptibility classes in different local units is given in Fig. 14.

**Figure 15:**
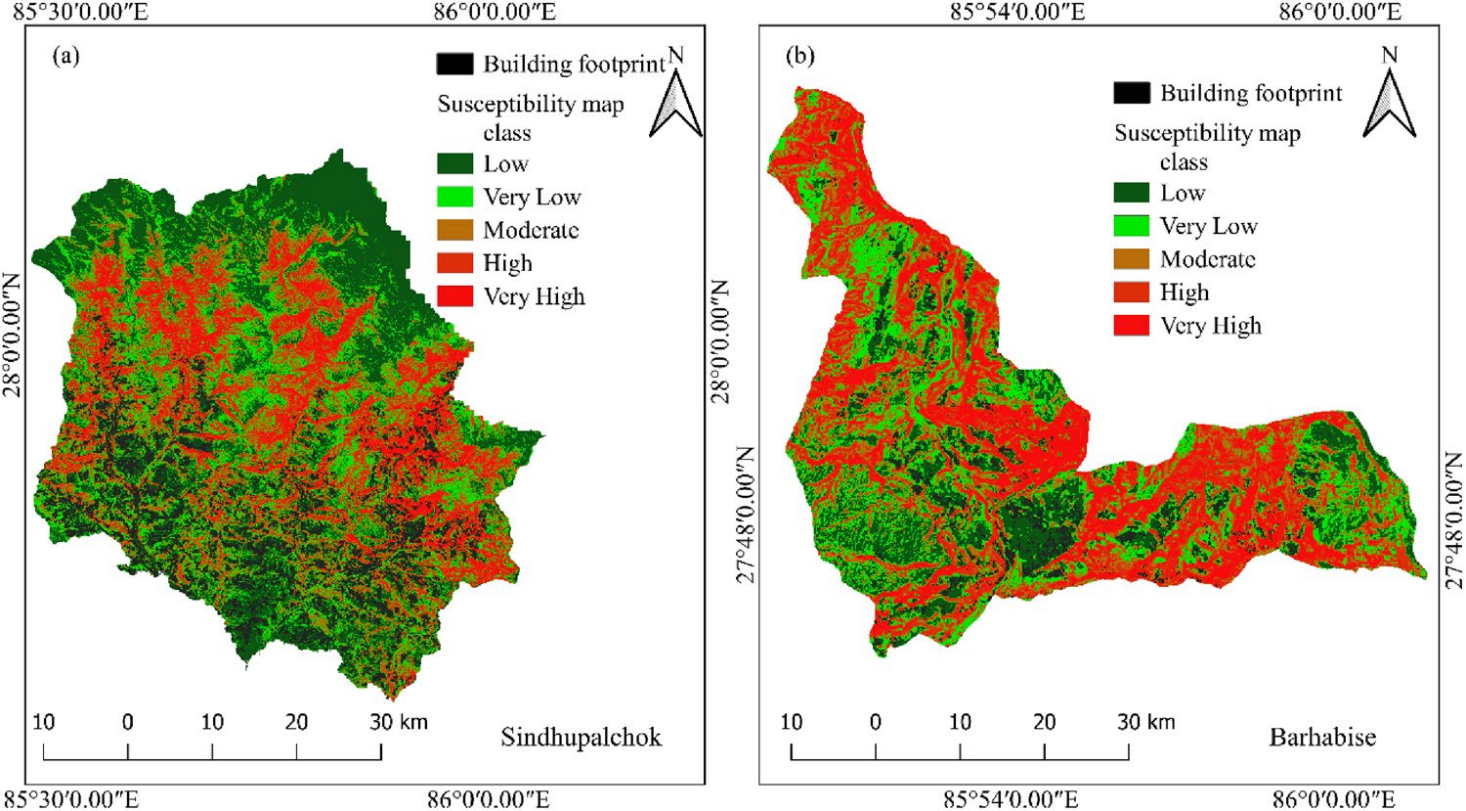
Most susceptible district (Sindhupalchok) and local unit (Barhabise) municipality of Sindhupalchok district.

From Fig. 14, it is observed that in Sindhupalchok district, the most susceptible local unit is Barhabise municipality, with 19.98% and 20.34% of its area under the high and very high susceptibility classes, respectively, inferring that more than 40% of the Municipality falls under the very high or high susceptibility class. The susceptibility map of the Sindhupalchowk district and the Barhabise municipality is shown in Fig. 15.

Figure 15 clearly shows the areas susceptible to high and very high landslides in and around Barhabise municipality. It is recommended to avoid these areas susceptible to high and very high landslides for developmental activities such as rural road construction, water resource management, hydropower projects, etc., and for settlements.

### Analysis of building exposure to co-seismic landslides

**Figure 16:**
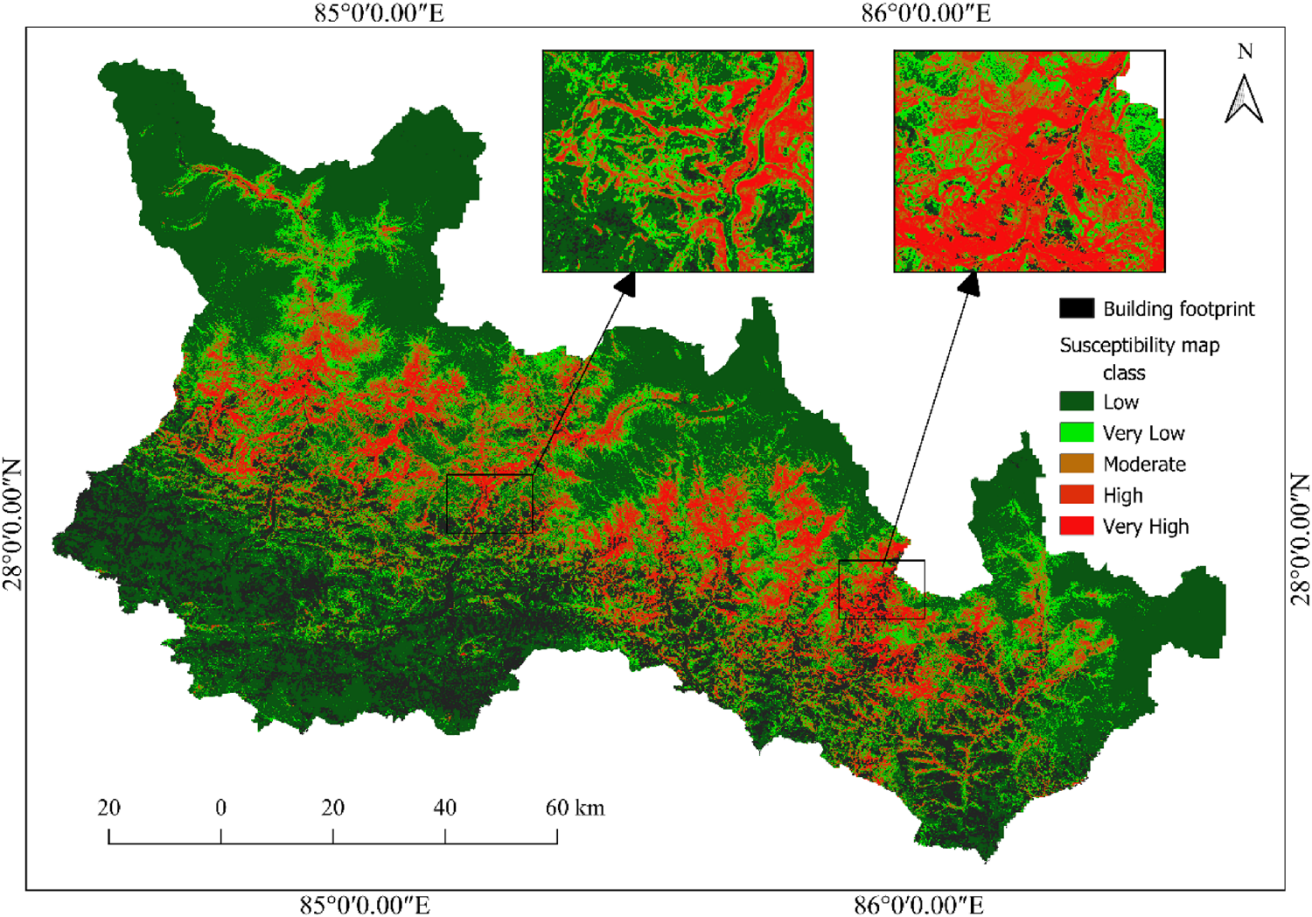
Building overlay on the co-seismic landslide susceptibility map.

**Figure 17:**
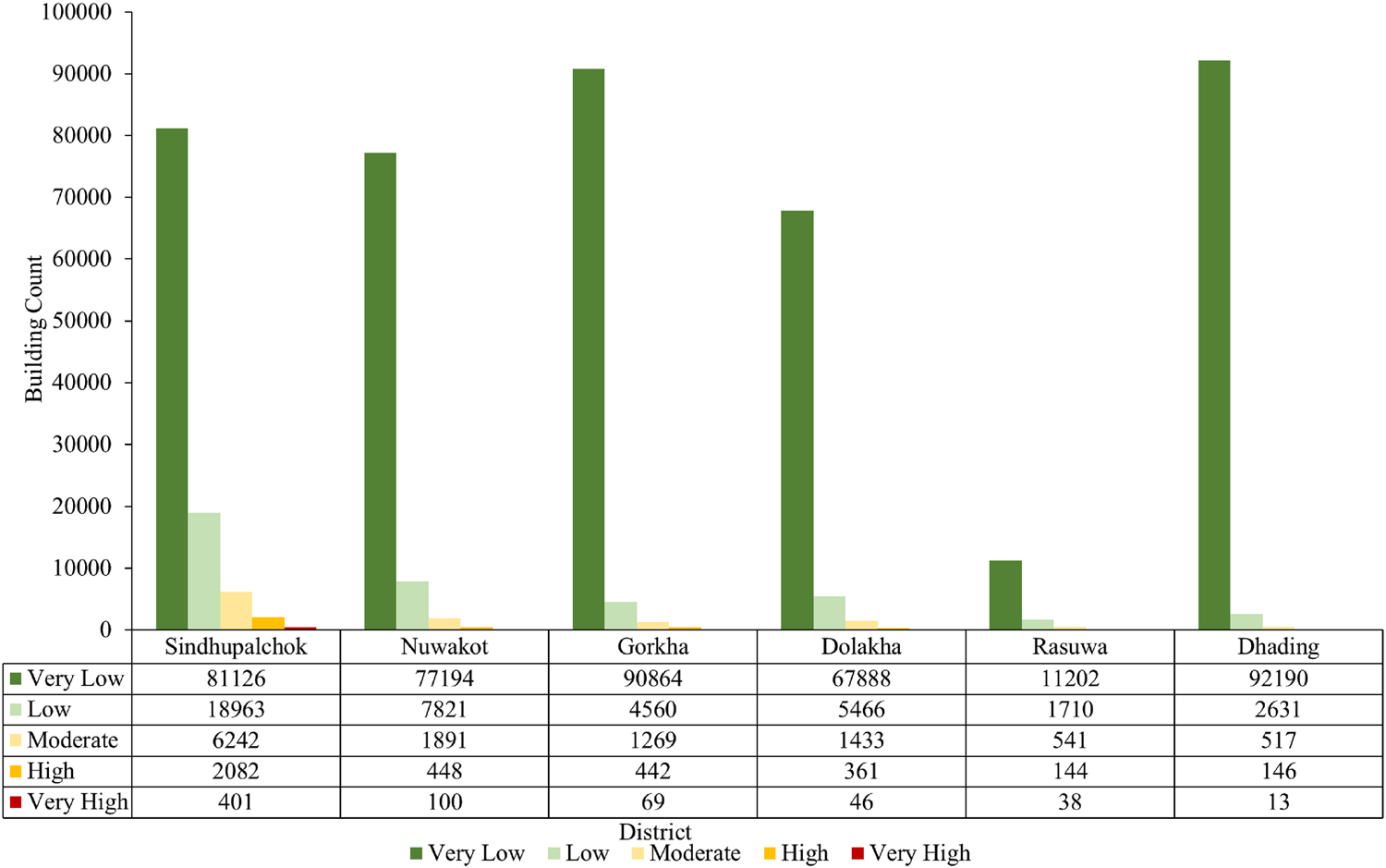
Building counts over the study area for each district.

To analyze the buildings exposed to co-seismic landslide susceptibility, a building footprint map is overlaid on the co-seismic susceptibility map, as shown in Fig. 16. By using the zonal statistics method in QGIS, the buildings in each susceptibility class are counted, and most of the buildings lie in the very low and low susceptibility classes. It is also observed that in the Sindhupalchok district, 2483 buildings are in very high and high susceptibility classes. For the Nuwakot and Gorkha districts, 548 and 510 buildings are located in the very high and high susceptibility classes, respectively. The lowest number of buildings, i.e., 159, are exposed to high and very high susceptibility classes in the Dhading district. Detailed information on the number of buildings in different susceptibility classes in each district is shown in Fig. 17.

**Figure 18:**
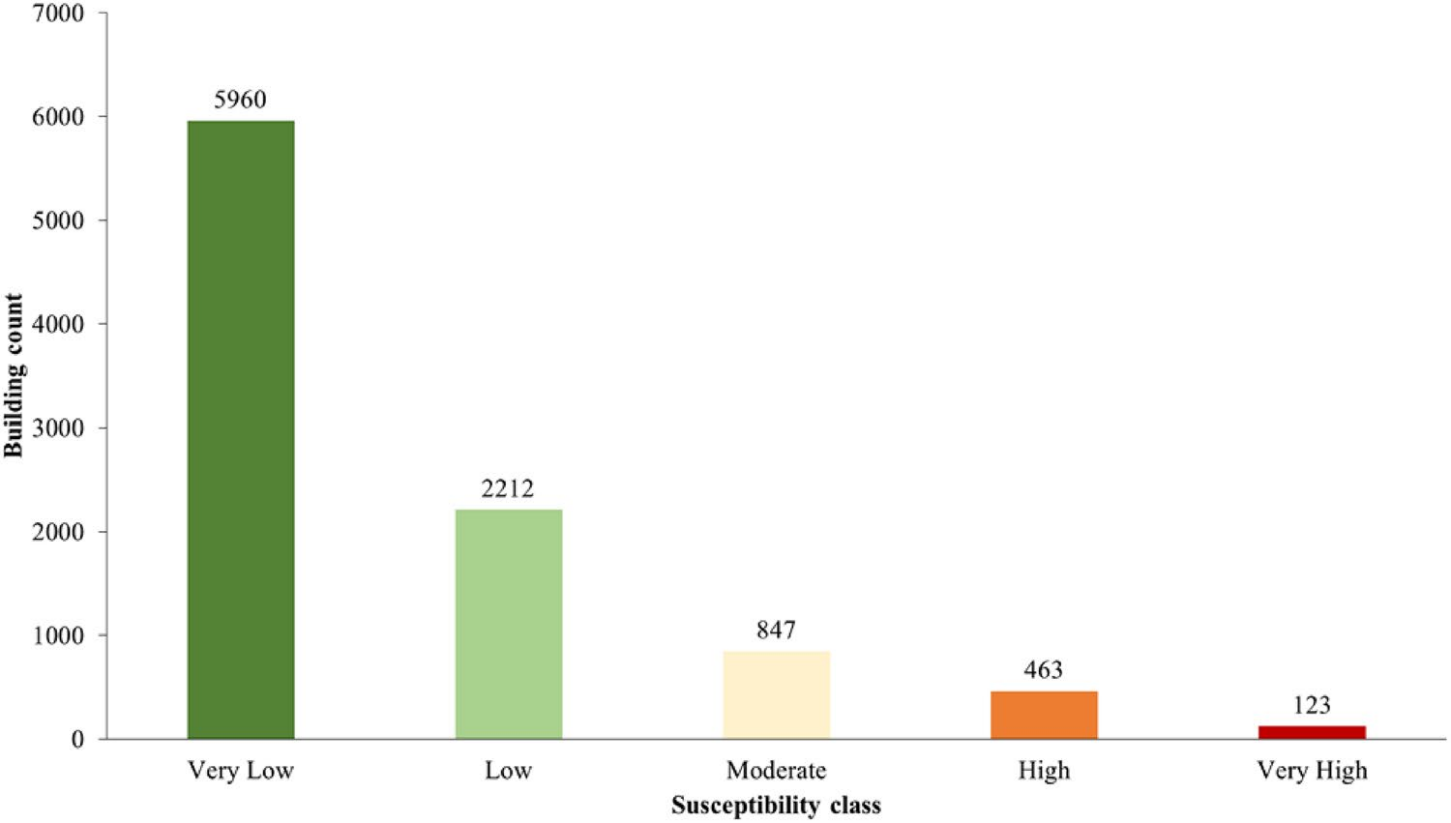
Building Count for Barhabise municipality.

In the Sindhupalchok district, 2483 buildings, or 3.06% of the total building count, fall into the “high” or “very high” classes. Similarly, 6.1% of the buildings in the Barhabise Municipality fall into these susceptibility classes (Fig. 18). The ratio is double that of the district, which is remarkably similar to the percentages in the region that fall into these susceptibility classes, namely, the district (24%) and the Barhabise (40%). According to this exposure distribution, as the area of high susceptibility increases, so does the building's exposure, posing significant risks to lives and property as well as long-term socioeconomic losses.

**Figure 19:**
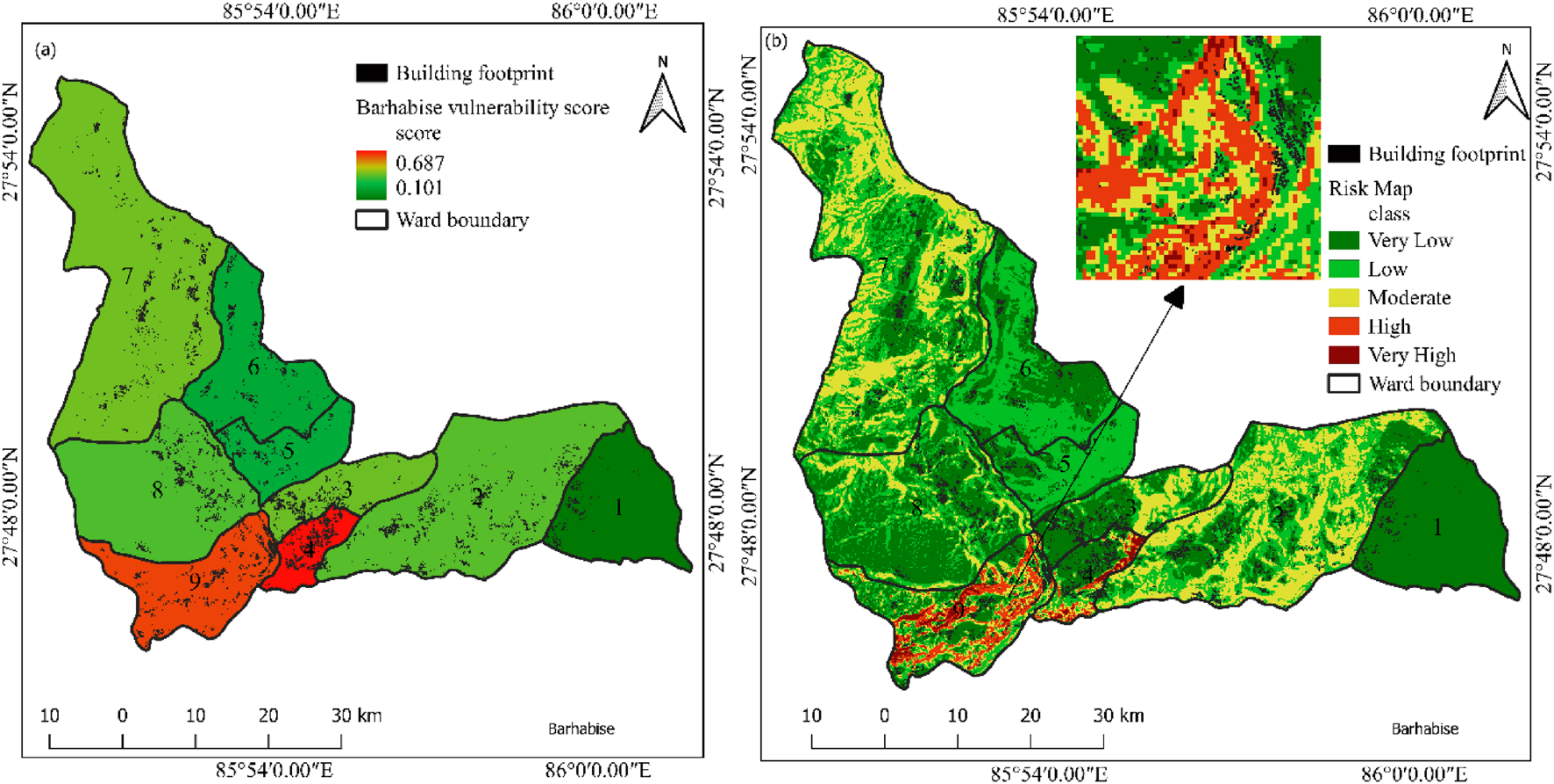
Vulnerability and risk map of Barhabise municipality with building footprint.

Ward level vulnerability mapping is carried out in Barhabise municipality. The data was acquired from national population and housing census 2021. The analysis was carried out following the method mentioned in “Vulnerability assessment” section above and the result is shown in Fig. 19.

**Figure 20:**
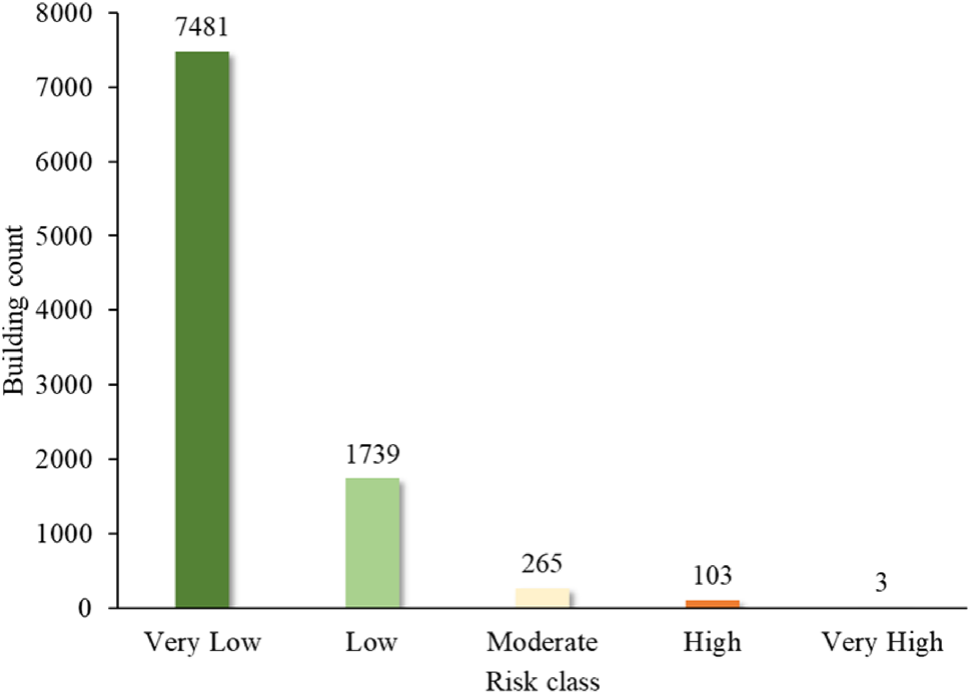
Building counts in different risk classes.

**Figure 21:**
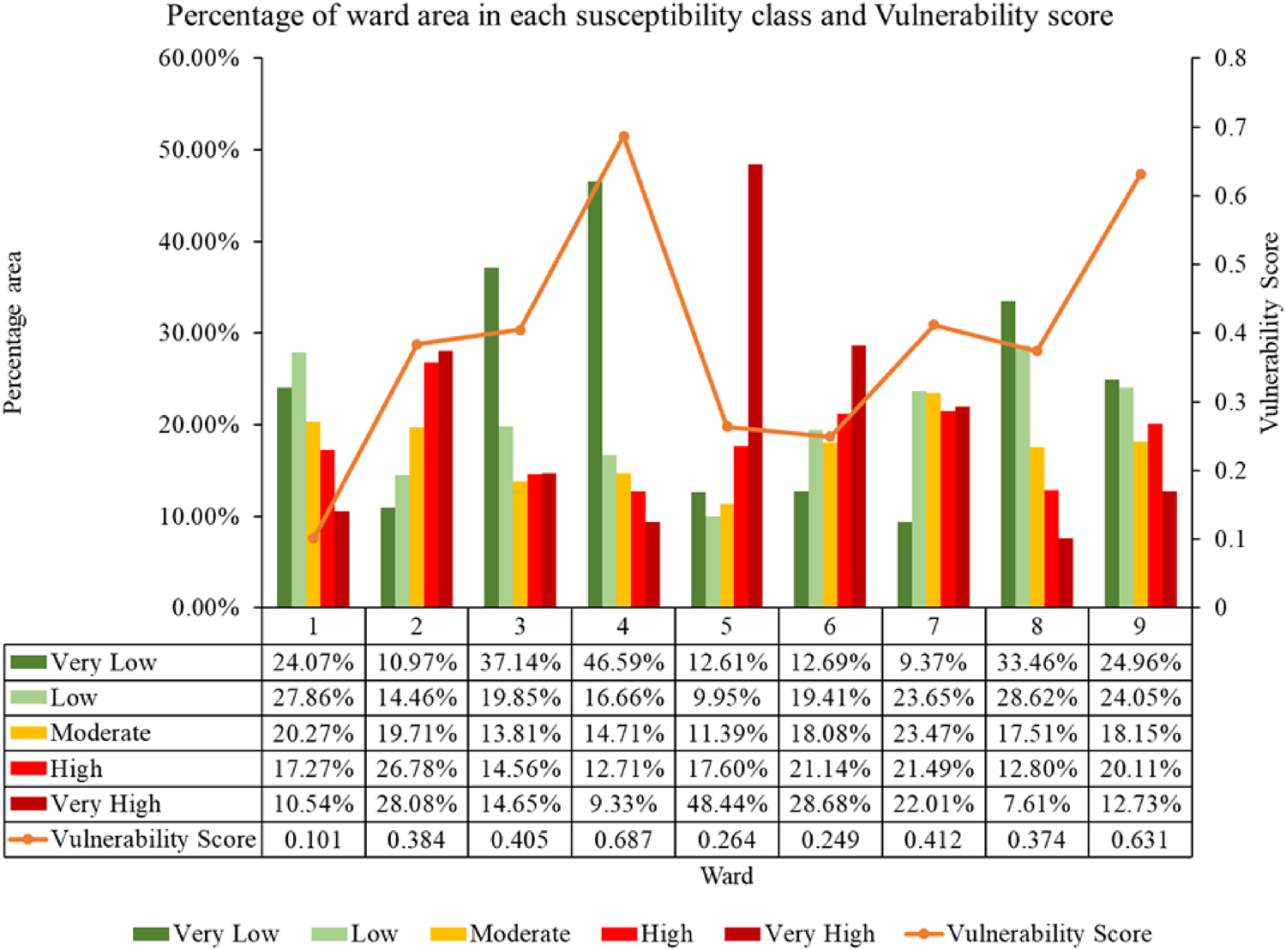
Vulnerability score with susceptibility class area in each ward of Barhabise municipality.

For Barhabise municipality, vulnerability score varies from 0.101 to 0.687. The risk map was classified in to five classes with equal interval. The risk map revealed that, the ward is located in high risk zone with 106 number of building exposed to high and very high-risk class. The detailed outcome of the vulnerability and risk assessment are shown in Figs. 20 and 21.

## Conclusion

Using Extremely Randomized Trees for analysis, the overall accuracy of the model is 87.2%, the area under the ROC curve is 0.94, the kappa coefficient is 0.744 and the RMSE value is 0.358, which indicates that the performance of the model is excellent with the considered causative factors. Therefore, district wise susceptibility shows that the Sindhupalchowk district is the most susceptible district, with 14.14% of the area under the high and 9.68% of the area under the very high susceptibility classes. Furthermore, the most susceptible local unit in Sindhupalchowk is Barhabise municipality, with 19.98% and 20.34% of its area under the high and very high susceptibility classes, respectively. To analyze the exposure of buildings to co-seismic landslide susceptibility, the building footprint map is overlayed on the landslide susceptibility map. From the analysis, the Sindhupalchowk district has the highest number, that is, 2483 buildings in high and very high susceptibility classes, and the Dhading district has the lowest number of buildings, that is, 159 buildings in high and very high susceptibility classes. Similarly, the most susceptible local unit is found as Barhabise Municipality, where nearly 40% of the area falls under the high and very high susceptibility classes. The building count that falls under these categories is 586, which is also the highest in a single local unit. While, the risk analysis using susceptibility mapping, considering socio-economic factors and structural vulnerability in the municipality, indicated that only 106 households, constituting 1.1% of the total 9591, were identified as high and very high risk.

Furthermore, till date, many studies have been conducted in Nepal for landslide susceptibility mapping that are focused on a small area, but this is the first study to evaluate co-seismic landslide susceptibility and building exposure analysis considering a large area using a machine learning approach. Similarly, local level risk mapping and exposure analysis is carried out in this study which can provide a critical and handy information from the policy makers to the local people understand the risk and possible threat imposed by the co-seismic landslide. This study can be a reference for further research on co-seismic susceptibility mapping and postseismic landslide hazard mitigation considering different earthquake scenarios in Nepal as well as throughout the world with conditioning factors similar to those in this study.

## Data Availability

The datasets used and/or analyzed during the current study available from the corresponding author on reasonable request.

## References

[1] Varnes DJ (1978). Slope movement types and processes [Tipos y procesos de movimiento de pendientes]. Landslides: Analysis and control. Transp. Res. Board Spec. Rep..

[2] Youssef AM, Pourghasemi HR (2021). Landslide susceptibility mapping using machine learning algorithms and comparison of their performance at Abha Basin, Asir Region, Saudi Arabia. Geosci. Front..

[3] Merghadi A (2020). Machine learning methods for landslide susceptibility studies: A comparative overview of algorithm performance. Earth Sci. Rev..

[4] KC D, Dangi H, Hu L (2022). assessing landslide susceptibility in the northern stretch of Arun Tectonic Window, Nepal. Civ. Eng..

[5] Zhou S, Fang L (2015). Support vector machine modeling of earthquake-induced landslides susceptibility in central part of Sichuan Province, China. Geoenviron. Disasters.

[6] Miao Z (2022). Integrating data modality and statistical learning methods for earthquake-induced landslide susceptibility mapping. Appl. Sci..

[7] Shahi YB (2022). Geological exploration, landslide characterization and susceptibility mapping at the boundary between two crystalline bodies in Jajarkot, Nepal. Geotechnics.

[8] Dahal BK, Dahal RK (2017). Landslide hazard map: Tool for optimization of low-cost mitigation. Geoenviron. Disasters.

[9] KC, D., Dangi, H., Naqvi, M. W. & Hu, L. Landslide mobilized debris flow at Kalli village in Achham, Nepal: A case study. Preprint at (2021).

[10] Kc D, Gautam K, Dangi H, Kadel S, Hu L (2022). Challenges in tunneling in the Himalayas: A survey of several prominent excavation projects in the Himalayan Mountain Range of South Asia. Geotechnics.

[11] Gabet EJ, Burbank DW, Putkonen JK, Pratt-Sitaula BA, Ojha T (2004). Rainfall thresholds for landsliding in the Himalayas of Nepal. Geomorphology.

[12] Dahal RK, Hasegawa S (2008). Representative rainfall thresholds for landslides in the Nepal Himalaya. Geomorphology.

[13] MOHA. Crisis to Resilience: Transforming Through Disaster Risk Reduction and Management. in *Position Paper Asia Pacific Ministerial Conference on Disaster Risk Reduction 2022 (APMCDRR-2022)* (2022).

[14] Roback K (2018). The size, distribution, and mobility of landslides caused by the 2015 Mw7.8 Gorkha earthquake, Nepal. Geomorphology.

[15] Bijukchhen SM, Kayastha P, Dhital MR (2013). A comparative evaluation of heuristic and bivariate statistical modelling for landslide susceptibility mappings in Ghurmi-Dhad Khola, east Nepal. Arab. J. Geosci..

[16] Ado M (2022). Landslide susceptibility mapping using machine learning: A literature survey. Remote Sens. (Basel).

[17] Zhao, B., Zhu, J., Hu, Y., Liu, Q. & Liu, Y. Mapping landslide sensitivity based on machine learning: A case study in Ankang City, Shaanxi Province, China. *Geofluids* **2022** (2022).

[18] Ngadisih N, Bhandary NP, Yatabe R, Dahal RK (2016). Logistic regression and artificial neural network models for mapping of regional-scale landslide susceptibility in volcanic mountains of West Java (Indonesia). AIP Conf. Proc..

[19] Zhang W, Li H, Han L, Chen L, Wang L (2022). Slope stability prediction using ensemble learning techniques: A case study in Yunyang County, Chongqing, China. J. Rock Mech. Geotech. Eng..

[20] Pourghasemi HR, Rahmati O (2018). Prediction of the landslide susceptibility: Which algorithm, which precision?. Catena (Amst).

[21] Pyakurel A, Dahal BK, Gautam D (2023). Does machine learning adequately predict earthquake induced landslides?. Soil Dyn. Earthq. Eng..

[22] Regmi AD (2014). Application of frequency ratio, statistical index, and weights-of-evidence models and their comparison in landslide susceptibility mapping in Central Nepal Himalaya. Arab. J. Geosci..

[23] Devkota KC (2013). Landslide susceptibility mapping using certainty factor, index of entropy and logistic regression models in GIS and their comparison at Mugling-Narayanghat road section in Nepal Himalaya. Natl. Hazards.

[24] Pokharel B, Alvioli M, Lim S (2021). Assessment of earthquake-induced landslide inventories and susceptibility maps using slope unit-based logistic regression and geospatial statistics. Sci. Rep..

[25] Gautam P, Kubota T, Aditian A (2021). Evaluating underlying causative factors for earthquake-induced landslides and landslide susceptibility mapping in Upper Indrawati Watershed, Nepal. Geoenviron. Disasters.

[26] Shrestha S, Kang TS, Suwal MK (2017). An ensemble model for co-seismic landslide susceptibility using GIS and random forest method. ISPRS Int. J. Geo-Inf..

[27] Shrestha S, Kang TS, Choi JC (2018). Assessment of co-seismic landslide susceptibility using LR and ANCOVA in Barpak region, Nepal. J. Earth Syst. Sci..

[28] Regmi AD, Dhital MR, Zhang JQ, Su LJ, Chen XQ (2016). Landslide susceptibility assessment of the region affected by the 25 April 2015 Gorkha earthquake of Nepal. J. Mt. Sci..

[29] Band SS (2020). Flash flood susceptibility modeling using new approaches of hybrid and ensemble tree-based machine learning algorithms. Remote Sens..

[30] Geurts P, Ernst D, Wehenkel L (2006). Extremely randomized trees. Mach. Learn..

[31] Sachdeva S, Kumar B (2022). Flood susceptibility mapping using extremely randomized trees for Assam 2020 floods. Ecol. Inform..

[32] UNDRR. Sendai Framework Terminology on Disaster Risk Reduction | UNDRR. https://www.undrr.org/drr-glossary/terminology.

[33] Perera ENC, Jayawardana DT, Ranagalage M, Dissanayake DMSLB, Wijenayaka HMDS (2020). Introduce a framework for landslide risk assessment using geospatial analysis: A case study from Kegalle District, Sri Lanka. Model. Earth Syst. Environ..

[34] Shah NA, Shafique M, Ishfaq M, Faisal K, Van der Meijde M (2023). Integrated approach for landslide risk assessment using geoinformation tools and field data in Hindukush mountain ranges, Northern Pakistan. Sustainability.

[35] Abbas N, Afsar S, Jan B, Sayla EA, Nawaz F (2022). GIS based model for the landslides risk assessment. A case study in Hunza-Nagar settlements, Gilgit-Baltistan, Pakistan. Environ. Chall..

[36] Bell R, Glade T (2004). Quantitative risk analysis for landslides & examples from Bíldudalur, NW-Iceland. Natl. Hazards Earth Syst. Sci..

[37] Althuwaynee OF, Pradhan B (2017). Semi-quantitative landslide risk assessment using GIS-based exposure analysis in Kuala Lumpur City. Geomat. Natl. Hazards Risk.

[38] Akgun A, Kıncal C, Pradhan B (2012). Application of remote sensing data and GIS for landslide risk assessment as an environmental threat to Izmir city (west Turkey). Environ. Monit. Assess.

[39] Quantum Geographic Information System. https://qgis.org/en/site/ (2023).

[40] Chen W, Pourghasemi HR, Zhao Z (2017). A GIS-based comparative study of Dempster-Shafer, logistic regression and artificial neural network models for landslide susceptibility mapping. Geocarto Int..

[41] Vakhshoori V, Zare M (2018). Is the ROC curve a reliable tool to compare the validity of landslide susceptibility maps?. Geomat. Natl. Hazards Risk.

[42] Elliott JR (2016). Himalayan megathrust geometry and relation to topography revealed by the Gorkha earthquake. Nat. Geosci..

[43] Rane PR, Vincent S (2022). Landslide susceptibility mapping using machine learning algorithms for Nainital, India. Eng. Sci..

[44] Dangi H, Bhattarai TN, Thapa PB (2019). An approach of preparing earthquake induced landslide hazard map: A case study of Nuwakot District, central Nepal. J. Nepal Geol. Soc..

[45] Skilodimou HD, Bathrellos GD, Koskeridou E, Soukis K, Rozos D (2018). Physical and anthropogenic factors related to landslide activity in the Northern Peloponnese, Greece. Land.

[46] Zhang T (2022). Evaluation of different machine learning models and novel deep learning-based algorithm for landslide susceptibility mapping. Geosci. Lett..

[47] Pradhan B, Lee S, Buchroithner MF (2010). Remote sensing and GIS-based landslide susceptibility analysis and its cross-validation in three test areas using a frequency ratio model. Photogramm. Fernerkund. Geoinf..

[48] Nakileza BR, Nedala S (2020). Topographic influence on landslides characteristics and implication for risk management in upper Manafwa catchment, Mt Elgon Uganda. Geoenviron. Disasters.

[49] Yalcin A, Bulut F (2007). Landslide susceptibility mapping using GIS and digital photogrammetric techniques: A case study from Ardesen (NE-Turkey). Natl. Hazards.

[50] Kanungo, D. P., Singh, R. & Dash, R. K. *Field Observations and Lessons Learnt from the 2018 Landslide Disasters in Idukki District, Kerala, India*.

[51] Rodríguez CE, Bommer JJ, Chandler RJ (1999). Earthquake-induced landslides: 1980–1997. Soil Dyn. Earthq. Eng..

[52] Keefer DK (1984). Landslides caused by earthquakes. Geol. Soc. Am. Bull..

[53] Lan JY (2022). A centrifuge study on the effect of the water cover on the ground motion of saturated marine sediments. Soil Dyn. Earthq. Eng..

[54] Dai FC (2011). Spatial distribution of landslides triggered by the 2008 Ms 8.0 Wenchuan earthquake, China. J. Asian Earth Sci..

[55] Qi S, Xu Q, Lan H, Zhang B, Liu J (2010). Spatial distribution analysis of landslides triggered by 2008.5.12 Wenchuan Earthquake, China. Eng. Geol..

[56] Zou Y (2022). Factors controlling the spatial distribution of coseismic landslides triggered by the Mw 6.1 Ludian earthquake in China. Eng. Geol..

[57] Grandin R (2015). Rupture process of the Mw = 7.9 2015 Gorkha earthquake (Nepal): Insights into Himalayan megathrust segmentation. Geophys. Res. Lett..

[58] Kobayashi T, Morishita Y, Yarai H (2015). Detailed crustal deformation and fault rupture of the 2015 Gorkha earthquake, Nepal, revealed from ScanSAR-based interferograms of ALOS-2. Earth Planets Space.

[59] Paudyal P, Dahal P, Bhandari P, Dahal BK (2023). Sustainable rural infrastructure: Guidelines for roadside slope excavation. Geoenviron. Disasters.

[60] McAdoo BG (2018). Roads and landslides in Nepal: How development affects environmental risk. Natl. Hazards Earth Syst. Sci..

[61] Karra, K. *et al*. Global land use/land cover with Sentinel-2 and deep learning, in *IGARSS 2021–2021 IEEE International Geoscience and Remote Sensing Symposium. IEEE* (2021).

[62] Esri. Sentinel-2 10m land use/land cover time series—Overview. https://www.arcgis.com/home/item.html?id=d3da5dd386d140cf93fc9ecbf8da5e31 (2022).

[63] Li F, Gong H, Chen B, Zhou C, Guo L (2020). Analysis of the contribution rate of the influencing factors to land subsidence in the eastern Beijing Plain, China based on extremely randomized trees (ERT) method. Remote Sens..

[64] Pham QB (2022). Predicting landslide susceptibility based on decision tree machine learning models under climate and land use changes. Geocarto Int..

[65] Raduszynski T, Numada M (2023). Measure and spatial identification of social vulnerability, exposure and risk to natural hazards in Japan using open data. Sci. Rep..

[66] ADRC. *Total Disaster Risk Management—Good Practice* (2005).

[67] NSO. Ward Report | National Population and and Housing Census 2021 Results by National Statistics Office. https://censusnepal.cbs.gov.np/results/downloads/ward (2023).

[68] Zhu Y, Tian D, Yan F (2020). Effectiveness of entropy weight method in decision-making. Math. Probl. Eng..

[69] Gao S (2020). Vulnerability assessment of marine economic system based on comprehensive index and catastrophe progression model. Ecosyst. Health Sustain..

[70] Jahan A, Edwards KL (2015). A state-of-the-art survey on the influence of normalization techniques in ranking: Improving the materials selection process in engineering design. Mater. Des..

[71] Vafaei N, Ribeiro RA, Camarinha-Matos LM (2018). Data normalisation techniques in decision making: Case study with TOPSIS method. Int. J. Inf. Decis. Sci..

[72] Xiao Y, Tang X, Li Y, Huang H, An BW (2022). Social vulnerability assessment of landslide disaster based on improved TOPSIS method: Case study of eleven small towns in China. Ecol Indic.

